# Academic reading format preferences and behaviors among university students worldwide: A comparative survey analysis

**DOI:** 10.1371/journal.pone.0197444

**Published:** 2018-05-30

**Authors:** Diane Mizrachi, Alicia M. Salaz, Serap Kurbanoglu, Joumana Boustany, on behalf of the ARFIS Research Group

**Affiliations:** 1 Charles E. Young Library, University of California, Los Angeles, California, United States of America; 2 University Libraries, Carnegie Mellon University, Doha, Qatar; 3 Information Management Department, Hacettepe University, Ankara, Turkey; 4 Institut Francilien d’Ingénierie des Services, Université Paris-Est Marne-la-Vallée, Paris, France; Fordham University, UNITED STATES

## Abstract

This study reports the descriptive and inferential statistical findings of a survey of academic reading format preferences and behaviors of 10,293 tertiary students worldwide. The study hypothesized that country-based differences in schooling systems, socioeconomic development, culture or other factors might have an influence on preferred formats, print or electronic, for academic reading, as well as the learning engagement behaviors of students. The main findings are that country of origin has little to no relationship with or effect on reading format preferences of university students, and that the broad majority of students worldwide prefer to read academic course materials in print. The majority of participants report better focus and retention of information presented in print formats, and more frequently prefer print for longer texts. Additional demographic and post-hoc analysis suggests that format preference has a small relationship with academic rank. The relationship between task demands, format preferences and reading comprehension are discussed. Additional outcomes and implications for the fields of education, psychology, computer science, information science and human-computer interaction are considered.

## Introduction

Instant information, communication, and connected information devices are now ubiquitous in many societies around the world. Today’s college students, in particular, have lived most or all of their lives in the modern information age. According to a Pew Research Study published in 2015, 92% of adolescents in the United States go online daily through their phones and other electronic devices [1]. Statistics from Australia, the UK and the United States indicate that children of all ages have steadily been increasing the amount of time spent each day engaged with electronic media over recent decades [2]. To quantify, the Kaiser Family Foundation has found that American youths aged 8–18 spend approximately 7.5 hours a day using electronic media [3], up from 6.43 hours in 2006 [2]. Based on these data, it might be natural to assume that college students who have grown up in this digital information environment would be more comfortable reading their academic materials electronically rather than in print. However, studies of students’ preferred format presentation show that this is frequently untrue.

Current knowledge about the suitability and impact of reading formats, whether print or electronic, for different purposes including learning, comprehension and usability is far from complete. In an era where e-textbook adoptions and blended/online learning components are expanding rapidly across higher education, there are indications that many university students today prefer to read academic materials in print and, even more pertinently for educators, believe they actually learn better from print materials. Higher education administrators and learning designers need to know who these readers are, the extent to which they use or prefer certain academic content formats, and the behavioral and learning implications of these preferences.

What we do know about differences in reading in print and electronic formats suffers from problems of scale and diversity. Studies on reading format preferences and behaviors to date have included anywhere from a few dozen to a few hundred participants, and the differing study designs and approaches make it a challenge to aggregate findings or distinguish clear patterns across study groups. Like most research across many subject fields, existing studies regarding print and digital reading preferences and behaviors also most commonly take data from participant samples in a short list of Western countries. While there are important reasons for this related to logistics and feasibility, data taken from geographically homogenous subsets of users leaves us with a limited ability to make reasonable inferences about human experience and behavior across much of the globe. Particularly where questions of innate versus environmental influences on the processing of text and cognition are concerned, we felt it important to identify and test assumptions about reading preferences with a larger and more diverse global sample than has been employed in previous studies. Variations in a sample that includes participants from countries with broadly different socioeconomic development levels, technological readiness levels, and schooling systems could be analyzed to help inform and develop models and hypotheses about “when, for whom, and for what purposes one mode of delivery (i.e., print or digital) might prove more beneficial than another”[4](p1009).

To that end, this study presents data about reading format preferences gathered from 10,293 college and university students in 21 countries worldwide using an original validated instrument. The Academic Reading Format International Study instrument (ARFIS) gathers self-reported reading format preferences, either print or electronic, and tests for the consistency of preferences across countries and languages. It also gathers data on the extent to which respondents say they utilize print and digital formats and their associated tools, and whether the language of publication (native or foreign to the respondent) influences their format preferences. It was developed and disseminated to answer the following research questions:

1. What format, print or electronic, do university students prefer for the majority of their academic course materials?
2. Do format preferences and behaviors vary by country?
3. Does the length of a text influence format preferences?
4. Is the language of the reading a confounding factor in evaluating format preferences?

The findings of this study are compared to previous findings and the implications for tertiary educators, publishers and technology developers are discussed.

This study defines and approaches reading in a particular manner. While we engage with text in various ways every day, many types of engagement do not amount to the type of “reading” we seek to understand in this study. For example, skimming, scanning, or quickly extracting factual data from news headlines, emails or text messages are activities and purposes that involve decoding and interpreting words and might be called “reading” in an everyday context, but for the purposes of this research are not included as an object of study. Rather, our focus is the type of reading typically conducted in a tertiary academic setting—engagement with text that involves “the retrieval of previously acquired [mental] schema to assist the processing and understanding of new unfamiliar information”[5](p55). In other words, learning from text.

In terms of “digital reading” or “e-reading”, then, we have in mind the activity of learning from text presented through any kind of electronic format, whether produced as a native electronic text, or scanned into a PDF from print and accessed via computer screen, dedicated e-reader, tablet or other digital hardware. This conception of reading as learning from text underpins the inquiry into how students at university prefer to read academic texts and course materials.

## Literature review

To date, a variety of reading preference studies have yielded mixed conclusions on the preferred format for reading among college and university students, with some finding a preference for print [6–11] and others finding a preference for electronic formats [12]. The differences in conclusions are difficult to explain given the range of tasks and approaches to inquiry found across studies, and explanatory theories or models of learning and behavior that can account for these findings and their differences are limited. Preference for electronic reading may occur in circumstances where texts are shorter; where reading purposes are different, as in reading for leisure or casual information consumption; or where a high value is placed on the affordability or portability afforded by digital texts. For instance, Wang and Bai found that undergraduate students tended to use e-books only for leisure reading and not for academic study[13]. Some researchers have suggested that environmental concerns with paper and printing drive preferences towards e-reading [14], and others have captured user attitudes towards print that seem deeply rooted in identity as a reader and nostalgia for hard copy reading [15] Still other studies have documented fundamental usability problems with e-formats which seem to drive print preference, such as difficulties with eyestrain [14], scrolling [16], and the usability of text engagement tools such as highlighting and notetaking [17]. Some have even suggested gender may play a role in format preference [18].

At the same time, cognitive studies over the last decade suggest that the presentation format of a text, either print or electronic, affects deep learning strategies, retention, and focus capabilities [19,18,20–22]. In a variety of experiments, print format has been found to offer an advantage for learning and remembering information conveyed in a text. In their recent systematic review of format-linked reading comprehension research, Singer and Alexander present evidence that these print advantages may be most pronounced where the processing and recall of more detailed, granular information is concerned, and when dealing with lengthier texts [4]. The learning and recall advantages evidenced in print reading may diminish when dealing with shorter texts or in circumstances where the broad themes or ideas of a text is all that needs to be understood or retained in memory [17]. The reasons for these differences are far from conclusive, but may have to do with how the layout and presentation of texts facilitates the construction of a mental map of the text and its overall structure [23], how the features or usability of contemporary e-reading platforms burden cognitive processes [16], or how the tactile features of print texts aid in memory encoding [24]. In the context of learning from text in a higher education setting, it is plausible that the perceived ease with which a format facilitates focus and memory might also drive preference. In at least some cases, however, stated user format preference and actual comprehension performance conflict [12], suggesting that respondents either are not aware of or do not always place high or sole importance on learning performance in making format decision.

While limited, certain theoretical perspectives have been presented as helpful lenses for interpreting these varied findings around user format preferences and identifying research directions. Mizrachi [25] suggests that Zipf’s principle of least effort [26] may help explain how and why readers report different format preferences in relation to different reading tasks and material types. This principle states that an actor will take the path of least effort or least resistance in order to achieve minimally acceptable results. In the context of selecting a format for reading, this principle predicts that readers would balance the ease, cost and convenience of electronic reading with the time and effort required to extract enough informational value from the text for the task at hand. If all that is required from the text is an enjoyable experience to pass the time while on vacation, as in reading a pulp fiction novel, a reader might well prefer an inexpensive, lightweight electronic version, as it gets the job done and the value placed by the reader on complex learning and retention of the information is low. If, however, a reader must concentrate carefully to learn deeply and recall granular levels of detail from texts, as when constructing a literature review for a thesis, they might find it less effortful to learn and accomplish the task using a print medium text. This is a broad principle which has been used to predict and explain a wide range of human behaviors across fields, including the information sciences, but is limited in that it lacks the specificity needed to account for the nuances of format preferences in different contexts.

Eshet-Alkalai and Geri [18] present a theory of information economics perspective which suggests that format preference is impacted by the economic value the reader places upon the reading, and can therefore vary depending on the context and desired outcome of the reading activity. It is a microeconomic perspective that looks at format choice through the lens of efficiency and game theory, considering the unique properties of information and the ways that information is valued as compared to other goods and services. This more domain-specific approach, which distinguishes informational products from other types of rational user or consumer-choice models, is useful to an extent for considering the factors driving user format choices. However, in academic settings, economic models can be limited in their explanatory power given the fact that the end-users of information, students, are not commonly the ones who choose readings, such as textbooks or other course materials, and as end-users, they may or may not directly pay for these materials. These factors undoubtedly distort the valuation of these resources and may introduce difficulty in quantifying or predicting perceived value.

These and other models and perspectives are helpful, but to the best of our knowledge there is not currently a cohesive theoretical basis for reconciling the sometimes contradictory findings outlined in this review. The uncertainty surrounding when, where and for whom print or digital reading formats better serve learning are of particular concern for educators and those involved with instructional design. Moreover, rapid changes occurring in the digital realm with regard to formats, platforms, costs, and resulting user behaviors, make a systematic and continuous data gathering program essential to understanding the use of, and improving the technology behind, digital formats for reading in the coming years.

Many of the ideas around which formats readers prefer and why can be explored further by analyzing format preference data from a broad international sample. For instance, ideas of personal identity as a reader and nostalgia for print reading are culturally linked and might be thought to be more prevalent in countries with a strong “book culture”. The socioeconomic development and technological readiness of countries might indirectly influence preferences by way of variables such as affordability and access to print or electronic formats. Device prevalence and experience levels linked to national educational systems and styles are potentially factors that correlate to greater print or electronic reading preferences. A broad, global dataset provides a rich opportunity for analysis of the factors driving format preferences in the context of higher education.

To that end, this study presents data from the first iteration of the academic reading format international study (ARFIS). The ARFIS instrument is designed to be used repeatedly to contribute empirical data to understandings of academic reading format preferences and behavior and their changes over time. It systematically gathers data in the form of reader self-reports that lend insight into how technological developments and instructional design approaches may be affecting and changing readers’ choices, in a manner that enables broad comparisons across an international sample. Data from this study presents a current snapshot of what the format preferences and self-reported behaviors among university students are, how they compare internationally, and how they compare to previously gathered data. This data lends insight into what students believe about their own learning from print versus digital formats, what factors may influence these preferences and what trends are occurring over time.

## Materials and methods

### Questionnaire design

The ARFIS questionnaire was developed based on a survey instrument used to examine UCLA undergraduates’ reading format preferences [25]. That instrument was piloted and refined before use, and subsequently extended by Mizrachi, Boustany, and Kurbanoglu into 16 Likert-style statements, six demographic questions, and an open-ended qualitative prompt for further comments. The instrument and study are designed to investigate a particular type of reader with a particular purpose—university students engaged in academic reading.

Students respond through an electronic platform, LimeSurvey, to eight statements related to format preferences both in general and under specific conditions, such as for shorter and longer readings, as well as five questions regarding their learning engagement behaviors across formats. Because in the global higher education landscape, students may study and be required to read texts in a lanaguage which is not their native language, three further questions seek to uncover any relationship between reading/publication language and format preferences. These statements, grouped by dimension, are:

**Dimension 1: Format Preference**
- I remember information from my course readings best when I read them from printed pages.
- It is more convenient to read my assigned readings electronically than to read them in print.*
- I prefer to have all my course materials in print format.
- If an assigned reading is 7 pages or more, I prefer to read it in print.
- If an assigned reading is less than 7 pages, I prefer to read it electronically.*
- I prefer electronic textbooks over print textbooks.
- I can focus on the material better when I read it in print.
- I prefer to read my course readings electronically.*

**Dimension 2: Learning Engagement (self-reported behaviors)**
- I usually highlight and notate my printed course readings.
- I usually highlight and annotate my electronic readings.*
- I am more likely to review my course readings (after I’ve read them at least once) when they are in print.
- I prefer to print out my course readings rather than read them electronically.
- I like to make digital copies of my printed course materials.*

**Dimension 3: Language**
- I prefer to read course readings which are in my native language elecronically rather than print.*
- I prefer reading foreign language material in print rather than electronic format.
- My preferred reading format, electronic or print, depends on the language of the reading.

Starred statements (*) are reverse-scored for analysis.

Demographic questions concerning country of origin, gender, age, visual limitations, academic major, and academic rank are employed to ensure diversity in the sample response but also to enable demographic analysis of response patterns. Because of the possible effects of electronic reading device features on user preference and reading comprehension implicated in prior studies[4], the instrument includes one statement concerning which devices participants use for electronic academic reading and allows multiple responses. Each Likert-style response item on the instrument offers five responses including Strongly Agree, Agree, Disagree, Strongly Disagree, and Neither Agree nor Disagree, and each item offers space for explanatory comments from participants. Likert-style items include opposing statements to attempt to minimize acquiescence bias in responses, although the balance of such statements is not perfectly even. Opposing question items are also constructed to avoid the use of double-negatives and use slightly different sentence constructions and terminology. These design considerations are intended to limit redundancy and the length of the instrument and maximize the number of complete responses.

The instrument items are assessed based on the responses for internal consistency using Cronbach’s alpha.

For Dimension 1, Format Preference, the subscale alpha based on 10293 valid responses across 8 items is 0.85. Each item in the scale contributes to a higher alpha for the scale as a whole and the corrected item-total correlations for each item range from 0.485 to 0.757. Therefore, the scale is reliable and internally consistent, and all items in this dimension have been retained in analysis.

Extending this scale to include statements pertaining to Dimension 2, Learning Engagement, improves the overall alpha scale to 0.87 over 10293 valid responses. However, item-by-item analysis reveals that the statement “I like to make digital copies of my printed course materials” is internally inconsistent (r = 0.202) with many of the other scale items and the scale total itself, and cannot be justified with face or theoretical validity. We have therefore dropped this item from the analysis, improving the final scale alpha to 0.882 across 12 items. The corrected item-total correlations for each of the remaining 12 items range from 0.391 to 0.711.

For Dimension 3, Language, the first two statements do not correlate particularly well with each other, with an alpha of 0.449 and Spearman-Brown coefficient of .451. For an indication of the extent to which participants believe language matters to their format preferences, we have focused primarily on the third statement (“My preferred reading format…depends on the language of the reading”) as an exploratory standalone indicator.

### Ethical compliance

The institutional review board authorized under the Office of Human Research Protection Program at the University of California, Los Angeles reviewed and approved the research plan. Approval was also granted by all participating institutions which require ethical clearance. Because data was gathered anonymously from participants with no personal identifiers collected in the process, no written consent was obtained from participants. Participants were informed of the study’s details and purposes through the LimeSurvey platform and completion of the survey is taken as implied consent.

### Participant recruitment

Researchers at partner institutions making up the international research team were recruited from personal and professional networks to help disseminate the questionnaire to university students worldwide, including translating the questionnaire from English into the local language of instruction if necessary. Student participants ranging from first-year through the doctoral level were recruited through their local institutions by email in 2014, 2015 and 2016. The study population is restricted to enrolled university students at any tertiary level. The purpose of the study is to assess the preferences and self-reported behaviors of readers engaged in learning from text, and the outcomes are intended to aid stakeholders in adult learning environments regarding educational design and practice. For this reason, the study is targeted at a population engaged in routine reading for learning—university students—and specifically addresses the use of academic course materials, not other types of reading. Participants from a broadly diverse international sample were sought in order to lend greater validity and reliability to the findings.

### Data analysis

Several of the researchers involved in data gathering have conducted separate independent analyses of subsets of the data gathered in their respective countries [6,27–32]. This study analyzes results from the complete international amalgamated quantitative data, relates descriptive and inferential statistical outcomes to show the worldwide trends uncovered in this study. Qualitative data gathered as part of this study is reserved for the use of local researchers only and is not reported here, in part due to the challenges involved in translating and aggregating these responses.

Quantitative data has been cleaned and analyzed twice using Excel and SPSS. Incomplete and unsubmitted responses were excluded from analysis. “Incomplete” refers to instances where participants left the survey early without responding to all format questions presented. Some of the questionnaires have missing gender data because the version of the questionnaire distributed did not present this item, and the otherwise complete responses of these participants were included and analyzed. Some responses include missing or unrealistic age data, because this item was presented as an open response. These otherwise complete responses are also included in the analysis. Descriptive statistics of participant responses to each question are presented using radial graphs for comparison. These descriptive graphics have been presented using the same scaling and increments in order to provide a broad comparative view of international trends. Descriptive statistical data is also presented in tabular form for ease of reading, and responses to Likert-style items have been collapsed from five categories to three representing those who agree to any extent with the statement, those who neither agree nor disagree, and those who disagree to any extent with the statement.

The 12-item format preference scale has been combined and presented using a median score for each participant. These scores have also been collapsed into the same three categories for reporting to give an overall descriptive indicator of format preference. The descriptive statistics address research questions about the nature of student preferences and the extent to which they self-report engagement in particular learning behaviors with text and the prevalence of types of digital devices they self-report using for academic reading. We have presented the findings here collectively as a scale total as well as item-by-item in order to facilitate a more granular view of the data and to more clearly demonstrate some of the differences across items.

Inferential statistical analysis of participant responses sought to confirm whether or not participants exhibit different format preferences or self-reported behavior patterns linked to country of origin and language of reading, and to estimate the significance of any such patterns. Data was analyzed using non-parametric statistical tests and the Likert-style responses have been treated as ordinal rather than scale data. Estimates of effect size for non-parametric tests [33] are given to illustrate the strength of differences between groups.

Demographic data about students’ gender, academic rank, and fields of study was gathered to assess the representativeness of the participant sample. Demographic data about students’ particular fields of study were reviewed and coded thematically according to Web of Science subject classifications in order to simplify the data and enable comparison.

Students in China responded to an inverted statement for item 10 of the instrument. The original survey statement “I prefer electronic textbooks over print,” was translated into the Chinese questionnaire as “I prefer print textbooks over electronic.” We have elected to retain this data in the analysis, and have inverted the original responses to this item in order to analyze them along with the larger dataset.

Post-hoc, randomized out-of-sample exploratory analysis of the results was also conducted to identify any potential patterns associated with a preference for print or digital reading of academic course materials that had not been previously hypothesized by the researchers. A random sample of 50% of the data (subset A) was selected for observation and analysis, and several new hypotheses were then tested against the other 50% of the data (subset B). Those newly generated hypotheses, supported and unsupported by subset B, are presented here to inform further research. Because of the size of the dataset, all inferential analysis was conducted looking at associations where p<0.01. No analysis was conducted below this threshold. It is our assumption, again based on the large size of the dataset, that true effects are likely to be observable in this range and unlikely to be missed, while spurious effects would more likely be observable in the .01 < p < .05 range.

## Results

Data reported in this analysis, gathered between 2014 and 2016, includes 10,293 complete survey responses from college and university students in 21 countries.

### Demographic analysis

Respondents are 67.47% female, 28.9% male, while 3.63% of respondents have no gender data. Male and female respondents across the full dataset showed statistically unique responses to the scale. Analysis by Mann-Whitney U generated test stastics *U* = 7,745,738, *W* = 12,172,538, *Z* = -20.704, with *η*^2^ = .043 and p < .0005. This suggests that the overall effect size is very small, and that 4.3% of the variability in responses is attributed to gender.

98.7% of participants range in age from 18 to 65, and 66.7% of all respondents (n = 6870) are 18–24 years old.

16.4% of respondents (n = 1686) indicated having some kind of visual impairment.

Enrollment status of respondents include first, second, third and fourth-year undergraduates (20.1%, 18.9%, 18.1%, 12.0% of respondents respectively), 21.9% master’s students, 5.7% doctoral students, and another 3.3% who report as “other.” Social science majors are the largest group of respondents (54.5%), followed by science majors (31.6%), and arts and humanities or other majors (13.9%). Table 1 lists the countries represented in this study, the number (n) and percentages of participants in each country sample, and the institutions from which the data was gathered.

**Table 1:** Representative institutions and country sample sizes.

Country	n	%	Institution(s)
**Bulgaria**	237	2.30	ULSIT, Sofia
**China**	1165	11.32	Sun Yat-Sen University; Peking University
**Croatia**	232	2.25	University of Zagreb
**Finland**	681	6.62	Oulu University
**France**	1630	15.84	Université Paris Descartes—IUT
**Israel**	135	1.31	Bar-Ilan University
**Italy**	1007	9.78	Università di Bologna
**Latvia**	1192	11.58	Latvijas Universitate
**Lebanon**	132	1.28	Lebanese University
**Moldova**	213	2.07	Academy of Economic Studies
**Norway**	1063	10.33	Bergen University, University of Science and Technology
**Peru**	208	2.02	Catholic University of Peru
**Portugal**	262	2.55	Oporto Polytechnic Institute
**Qatar**	105	1.02	University College London
**Romania**	188	1.83	Transylvania University
**Slovenia**	260	2.53	University of Ljubliana
**Switzerland**	170	1.65	Haute Ecole de Gestion
**Turkey**	214	2.08	Hacettepe University
**United Arab Emirates**	130	1.26	Khalifa University
**United Kingdom**	696	6.76	City University; London School of Economics and Political Science (LSE); University of Kent
**United States**	373	3.62	University of California Los Angeles
**Total**	**10,293**	**100**	

Participants reported which device(s) they use to read electronic academic material, and laptops are the most common answer among respondents (80.9%). Phones (36.83%), desktops (30.54%) and tablets (28.43%) are also popular devices. 4.34% of respondents report that they do not read material electronically. One finding of interest is that Chinese students’ use of phones for reading (73.7%) is double the median percentage of the amalgamated group (36.83%). Chinese participants also featured the highest percentages for use of tablets, e-readers and audio applications. This could reflect a broader integration of electronic technology in the Chinese educational culture, even though their participant format preferences do not vary to a large statistical effect from the rest of the respondents.

**Fig 1:**
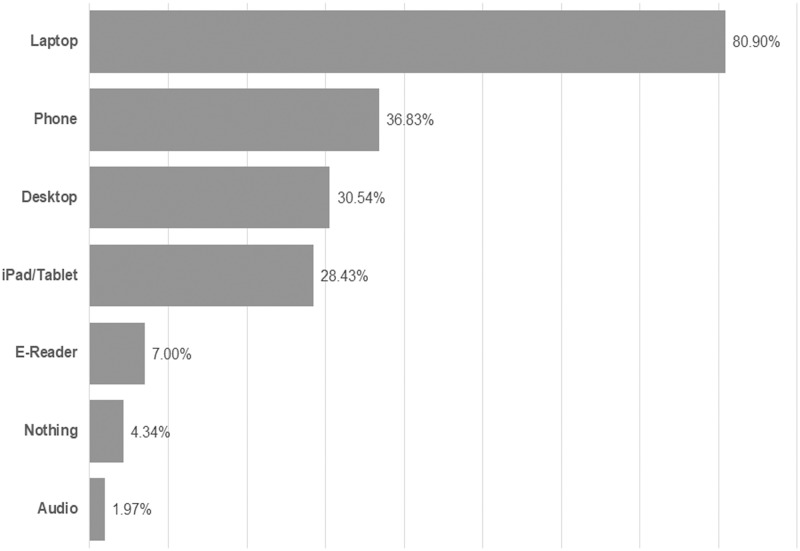
Reported usage of various e-reading devices among respondents (n = 10293).

Fig 1 displays the amalgamated totals for device usage and Table 2 shows the ranges of country responses from minimum to maximum.

**Table 2:** Ranges of country responses to device usage.

Device	Minimum	Maximum
Laptop	59.2% (Moldova)	91.5% (UAE)
Phone	20.1% (Norway)	73.7% (China)
Desktop	15.8% (U.S.)	53.5% (Moldova)
Tablet	14.6–7% (Slovenia, Croatia)	40.4% (China)
E-reader	1.5% (UAE)	23.7% (China)
w/Audio	0.0% (Portugal, Switzerland, UAE)	7.6% (China)
None/ Don’t read e-format	0.7% (Israel)	9.4% (France)

### Research questions

#### R1. What format, print or electronic, do university students prefer for the majority of their academic course materials?

The 12-item scale results show that overall, 78.44% of the 10,293 respondents prefer print format for reading academic course materials; 10.04% prefer electronic format; and 11.52% do not express a preference either way. Participant responses show more diversity when asked whether they prefer electronic format for shorter readings. Attitudes towards electronic textbooks are less favorable than print textbooks. Respondents believe that they focus and remember better when reading print, and that they are more likely to employ important learning engagement strategies such as highlighting and annotating, in their favored formats. 74.13% of respondents agree or strongly agree that they are more likely to review or revisit readings in print format.

#### R2. Do format preferences and behaviors vary by country?

**Fig 2:**
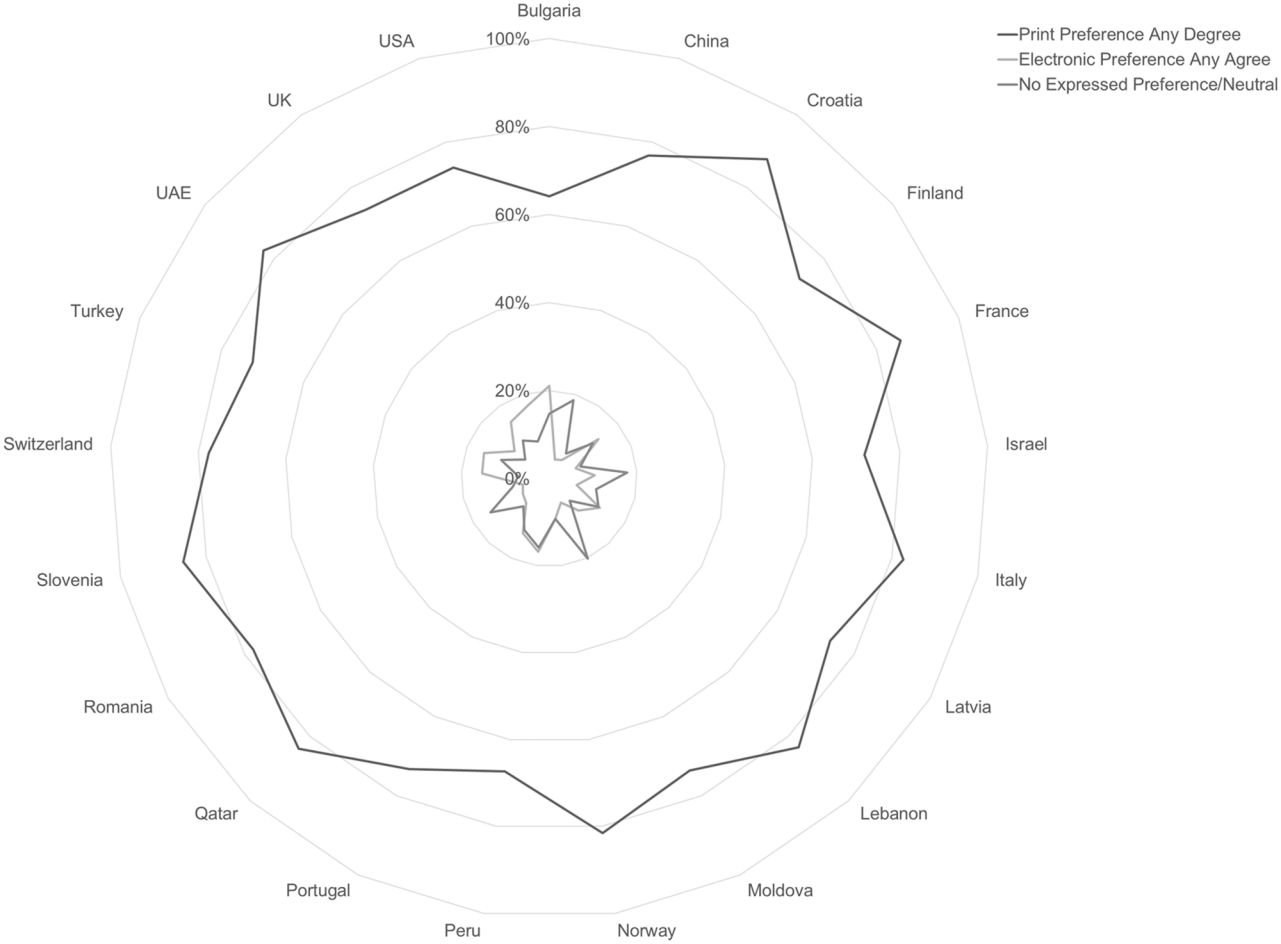
Format preferences by country, scale total, n = 10,293.

Overall response patterns regarding preference for print format for academic course readings are consistent worldwide, with large majorities in every country sample reporting that they prefer print format for academic course materials (Fig 2). Participants reported beliefs about their ability to focus and remember information by format show consistent majorities in favor of print in all countries studied, but small significant differences in rates of preference by country at p < .0005 are present.

While majorities of respondents in all countries showed preference for print formats across the ARFIS items, some small differences in the scale response distributions between countries exist. Analysis by Kruskal-Wallis H test found statistically significant similarities and differences among certain subgroups of countries. Test statistics from the data shown in Table 3, show results from the 12-item format preference scale are z = 730.269, *p* < .0005, with mean ranks ranging from 3608 (United States) to 6365 (Croatia). The null hypothesis, that country results will be the same, is rejected. The overall effect size is small, with an η^2^ of .069 which indicates that 6.9% of the variability in responses can be attributed to the country of origin, and an E^2^ effect size estimate of .071 This does not reach the threshold of 0.1 for what would be considered a small effect size [34]. Therefore, this effect can be considered very small.

**Table 3:** Homogenous subsets by country, n = 10,293.

		Subset								
		1	2	3	4	5	6	7	8	9
**Sample**^a^	**Bulgaria**	4030								
	**Israel**	4200	4200							
	**China**	4213	4213							
	**Turkey**	4445	4445	4445						
	**Portugal**	4445	4445	4445						
	**Moldova**	4456	4456	4456						
	**Peru**	4554	4554	4554	4554					
	**Latvia**		4652	4652	4652					
	**Lebanon**		4661	4661	4661	4661				
	**Finland**			4817	4817	4817				
	**USA**			5006	5006	5006	5006			
	**UK**				5152	5152	5152	5152		
	**Romania**					5217	5217	5217	5217	
	**Norway**						5383	5383	5383	
	**Italy**						5452	5452	5452	
	**Slovenia**						5480	5480	5480	
	**Switzerland**						5507	5507	5507	5507
	**UAE**							6059	6059	6059
	**Qatar**								6089	6089
	**France**									6255
	**Croatia**									6319
**Test Statistic**		6.91	14.93	10.092	15.035	7.042	10.363	13.628	15.092	9.598
**Sig. (2-sided test)**		0.329	0.037	0.183	0.01	0.134	0.11	0.034	0.02	0.048
**Adjusted Sig. (2-sided test)**		0.715	0.098	0.427	0.037	0.468	0.307	0.103	0.06	0.194

^a^Mean ranks rounded to the nearest integer. Subsets are based on asymptotic significances. The significance level is .01. Larger means show greater intensity of preference for print.

The findings point to broad consistency across countries in terms of favorability towards print for academic reading.

**Fig 3:**
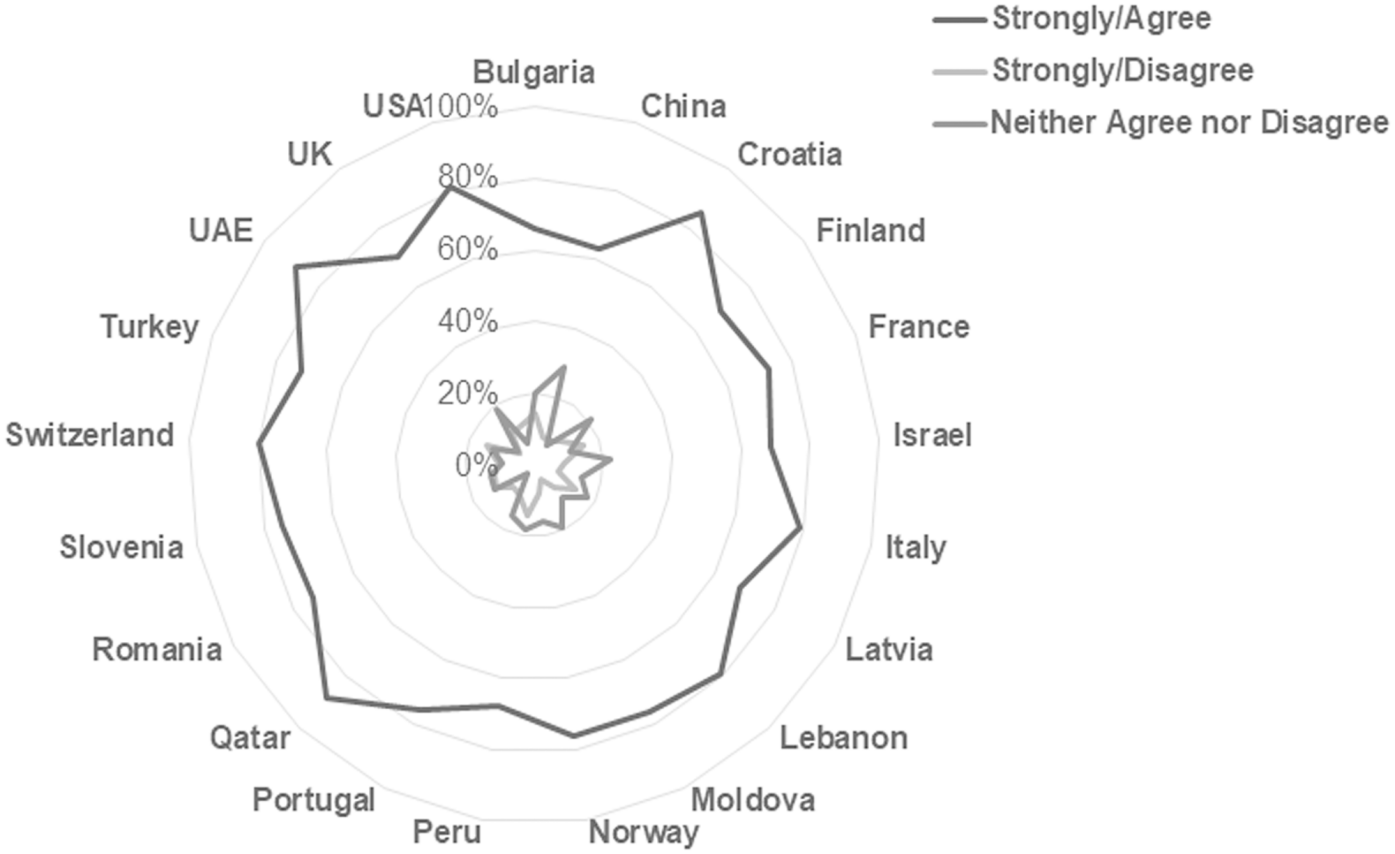
Responses to the statement “I remember information from my course readings best when I read them from printed pages” reported by country. [n = 10293; Agree/Strongly Agree n = 7450, 72.37%; Neither Agree nor Disagree n = 1687, 16.3%; Disagree/Strongly Disagree n = 1156, 11.23%].

**Fig 4:**
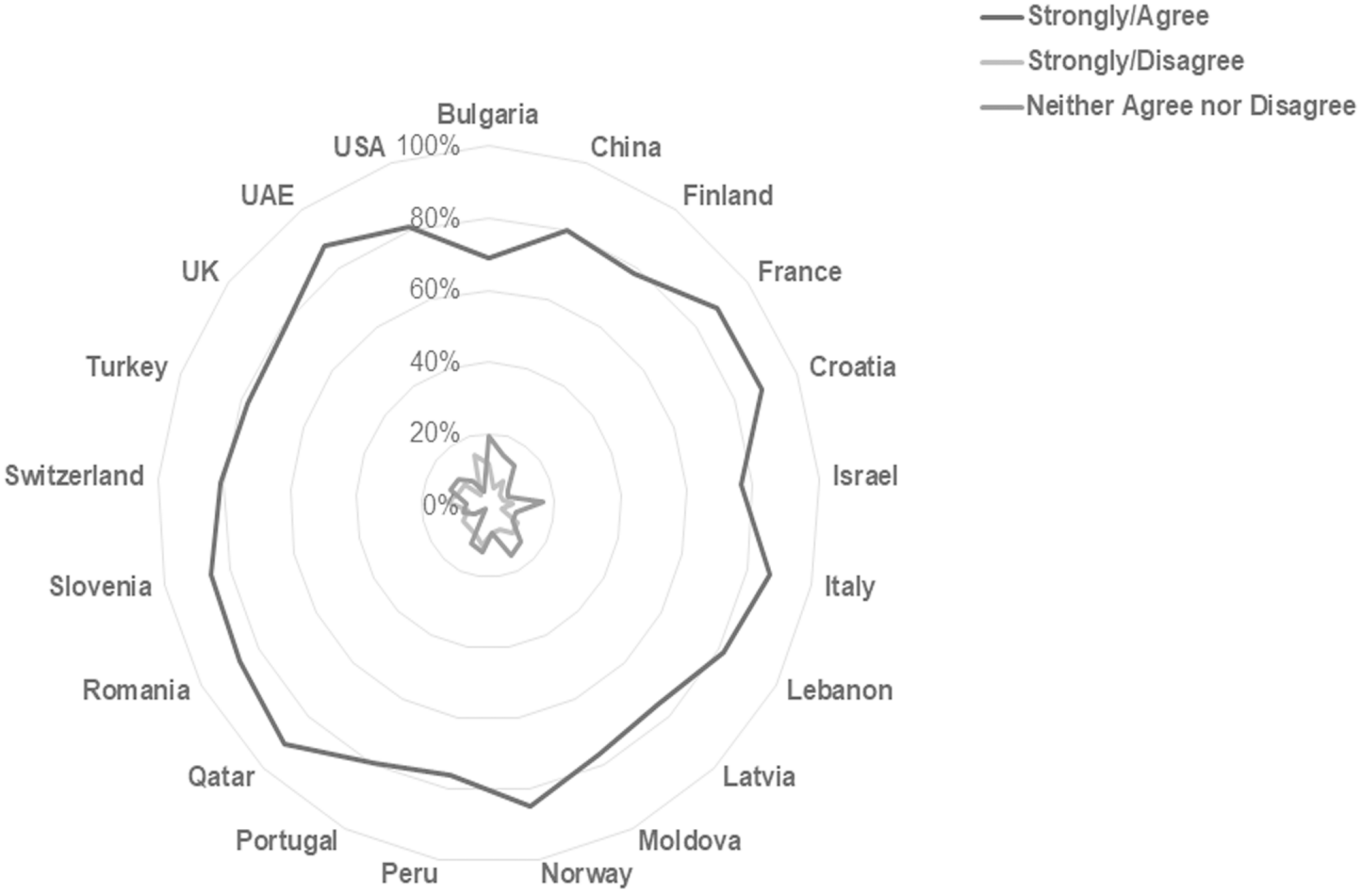
Responses to the statement “I can focus on the material better when I read it in print” reported by country. [n = 10293; Agree/Strongly Agree n = 8442, 82.02%; Neither Agree nor Disagree n = 1084, 10.53%; Disagree/Strongly Disagree n = 767, 7.45%].

This international consistency is more apparent in some individual scale item responses than in others. Findings in this study show that worldwide students believe print format is more conducive to remembering material, focusing on material, and the use of learning strategies such as highlighting, annotating and reviewing readings. In total, 72.37% of respondents agree or strongly agree that they remember information best from print sources, and 82.02% agree or strongly agree that they focus best with printed material. Figs 3 and 4 illustrate the consistency of response on these items across national subgroups. Tables 4 and 5 list the percentages by country for the respective statements.

**Table 4:** Responses to the statement “I remember information from my course readings best when I read them from printed pages” in percentages by country.

Country	n	% Agree/ Strongly Agree	% Disagree/ Strongly Disagree	% Neither Agree nor Disagree
**Bulgaria**	237	66%	14.3%	19.7%
**China**	1165	63.4%	8.1%	28.5%
**Croatia**	232	85.3%	8.2%	6.5%
**Finland**	681	68.6%	10.7%	20.7%
**France**	1630	72.8%	15.7%	11.4%
**Israel**	135	68.7%	9%	22.4%
**Italy**	1007	79.1%	7.1%	13.8%
**Latvia**	1192	68.8%	13.6%	17.5%
**Lebanon**	132	79.2%	8%	12.8%
**Moldova**	213	76.5%	4.2%	19.3%
**Norway**	1063	76.4%	7.6%	16%
**Peru**	208	67.7%	13.7%	18.6%
**Portugal**	262	75.5%	9.2%	15.3%
**Qatar**	105	82.9%	6.7%	10.5%
**Romania**	188	74.4%	13%	12.5%
**Slovenia**	260	75%	12.2%	12.9%
**Switzerland**	170	80.8%	10.9%	8.3%
**Turkey**	214	72.6%	15.1%	12.3%
**United Arab Emirates**	130	88.5%	5.4%	6.2%
**United Kingdom**	696	70%	11.1%	19%
**United States**	373	81.4%	11.7%	6.8%
**Total**	**10,293**			

**Table 5:** Responses by country to the statement “I can focus on the material better when I read it in print”.

Country	n	% Agree/ Strongly Agree	% Disagree /Strongly Disagree	% Neither Agree nor Disagree
**Bulgaria**	237	69%	11.8%	19.3%
**China**	1165	80.4%	5%	14.6%
**Croatia**	232	88.7%	4.7%	6.5%
**Finland**	681	78.2%	8.3%	13.5%
**France**	1630	88.1%	4.7%	7.1%
**Israel**	135	76.1%	7.4%	16.4%
**Italy**	1007	86.7%	4.5%	8.8%
**Latvia**	1192	75.4%	10.6%	14%
**Lebanon**	132	80.8%	10.4%	8.8%
**Moldova**	213	76.8%	7.5%	15.6%
**Norway**	1063	84.9%	7.3%	7.8%
**Peru**	208	75%	10.8%	14.2%
**Portugal**	262	79.7%	8.4%	11.9%
**Qatar**	105	84.8%	6.7%	8.6%
**Romania**	188	87%	8.7%	4.3%
**Slovenia**	260	85.9%	6.6%	7.4%
**Switzerland**	170	80.8%	12.9%	6.4%
**Turkey**	214	78.3%	9.9%	11.8%
**United Arab Emirates**	130	87.7%	3.9%	8.5%
**United Kingdom**	696	79%	9.3%	11.6%
**United States**	373	81.4%	14.2%	4.4%
**Total**	**10,293**			

**Fig 5:**
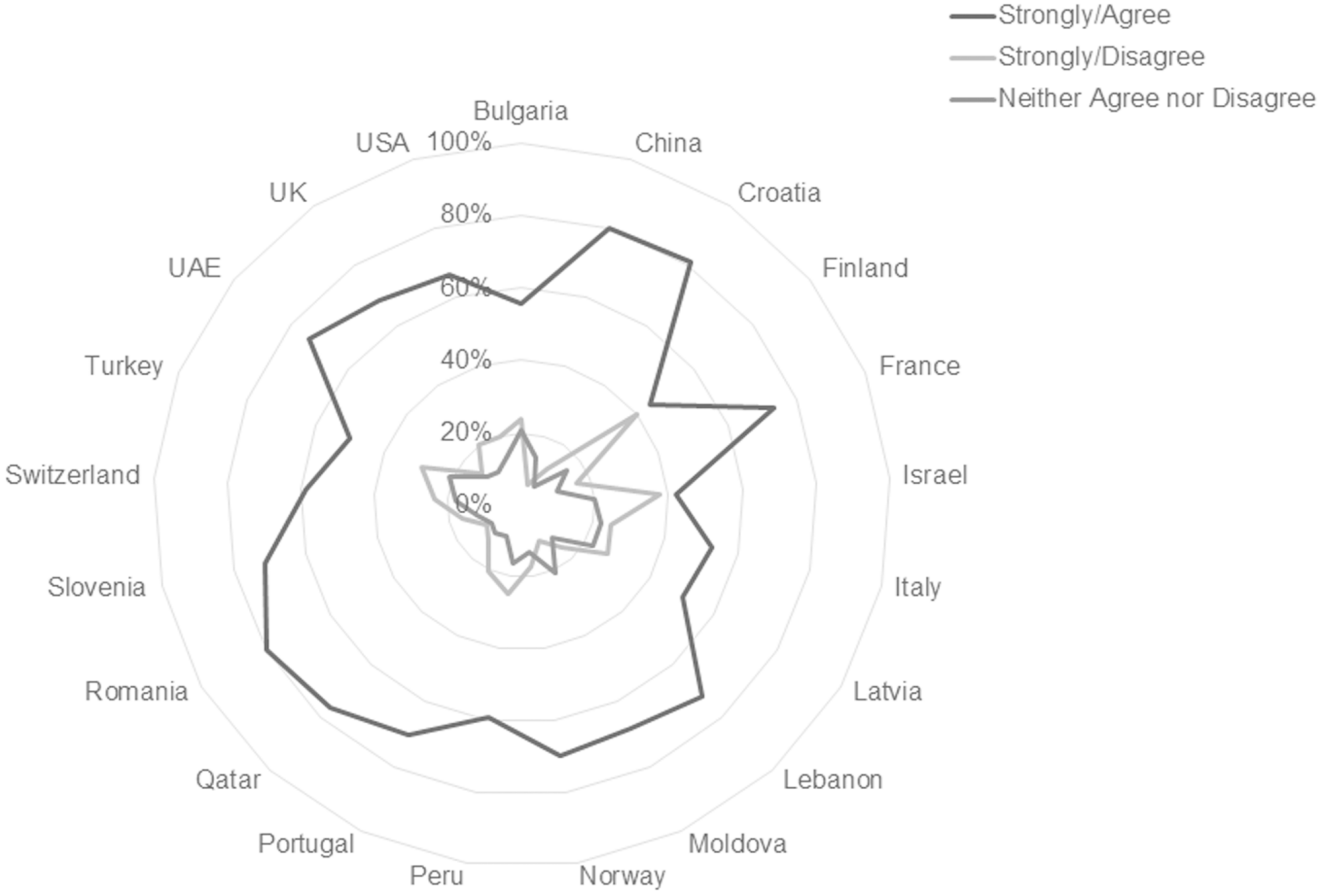
Responses to the statement “I prefer to have all my course materials in print format” reported by country. [n = 10293; Agree/Strongly Agree n = 6687, 64.97%; Neither Agree nor Disagree n = 1550, 15.05%; Disagree/Strongly Disagree n = 2056, 19.97%].

Across other single response items, more variation across national subgroups is perceptible. For example, Fig 5 and Table 6 show that the percentages of students in Finland and Israel agreeing and disagreeing that they prefer all their course materials in print differ by only 4.1% and 3.8% respectively, whereas much broader net favorable responses for print can be seen in other country responses.

**Table 6:** Responses by country to the statement “I prefer to have all my course materials in print format”.

Country	n	% Agree/ Strongly Agree	% Disagree/ Strongly Disagree	% Neither Agree nor Disagree
**Bulgaria**	237	55.9%	23.5%	20.6%
**China**	1165	80.1%	6.1%	13.8%
**Croatia**	232	81.4%	12.1%	6.5%
**Finland**	681	44.5%	40.4%	15.2%
**France**	1630	73.8%	16.3%	9.9%
**Israel**	135	41.8%	38%	20.1%
**Italy**	1007	52.7%	25.2%	22%
**Latvia**	1192	50.6%	26.9%	22.4%
**Lebanon**	132	71.2%	16%	12.8%
**Moldova**	213	68.4%	10.9%	20.8%
**Norway**	1063	69.7%	17.2%	13.1%
**Peru**	208	60.8%	22.6%	16.7%
**Portugal**	262	70.2%	20.3%	9.6%
**Qatar**	105	66.2%	13.3%	10.5%
**Romania**	188	79.9%	10.3%	9.8%
**Slovenia**	260	71.5%	16.8%	11.7%
**Switzerland**	170	60.2%	22.4%	17.3%
**Turkey**	214	50%	29.3%	20.8%
**United Arab Emirates**	130	73.9%	13.9%	12.3%
**United Kingdom**	696	68.3%	20.1%	11.3%
**United States**	373	66.9%	19.4%	13.7%
**Total**	**10,293**			

**Fig 6:**
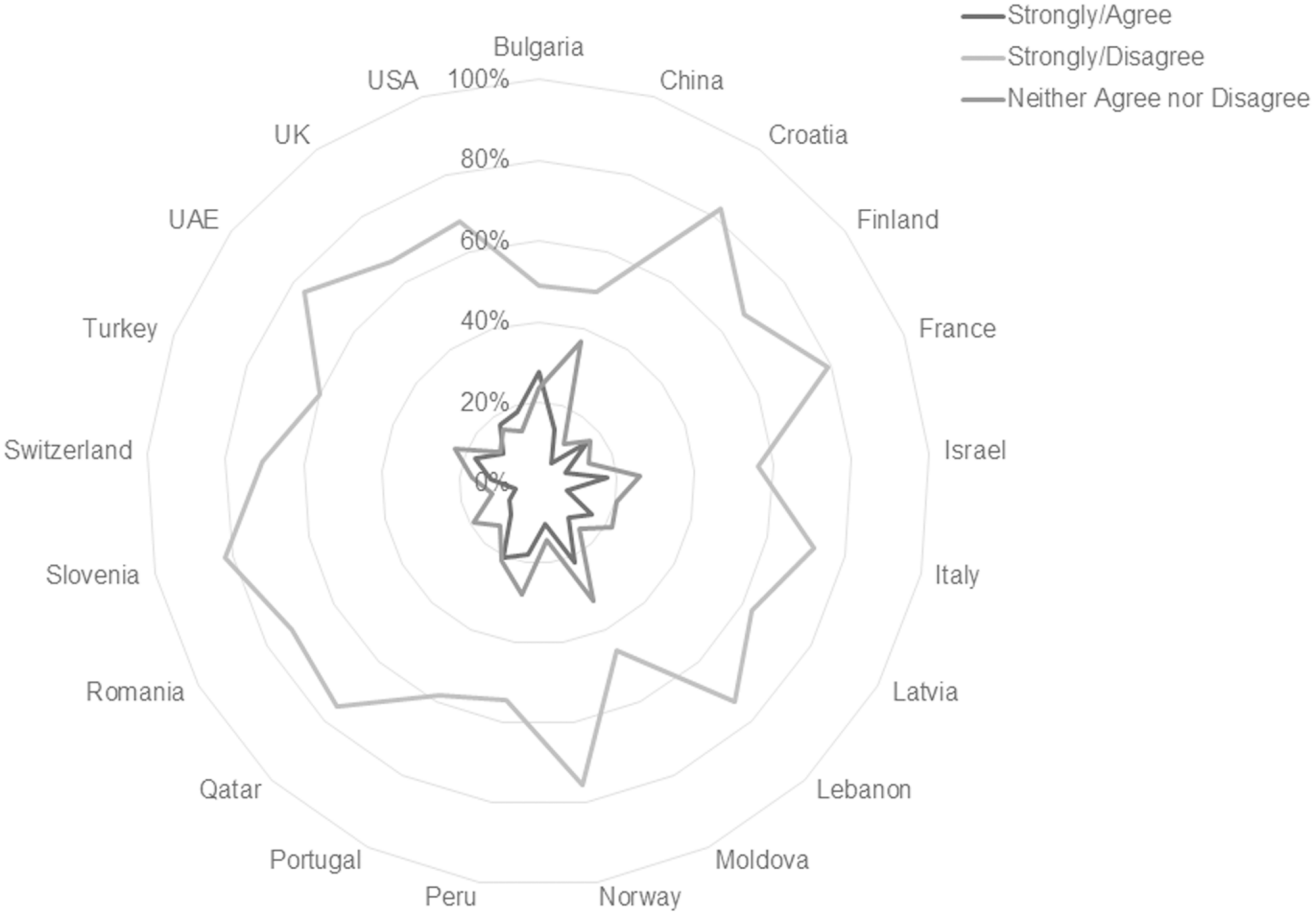
Responses to the statement “I prefer to read my course readings electronically” reported by country. [n = 10293; Agree/Strongly Agree n = 1320, 12.82%; Neither Agree nor Disagree n = 2039, 19.8%; Disagree/Strongly Disagree n = 6934, 67.37%].

Fig 6 and Table 7 show responses to the inverse of this question, which appear to be more uniform across countries.

**Table 7:** Responses by country to the statement “I prefer to read my course readings electronically”.

Country	n	% Agree/ Strongly Agree	% Disagree/ Strongly Disagree	% Neither Agree nor Disagree
**Bulgaria**	237	26.8%	49.1%	26.8%
**China**	1165	13.6%	49.5%	13.6%
**Croatia**	232	6%	82.2%	6%
**Finland**	681	16.1%	66.6%	16.1%
**France**	1630	7.4%	79.3%	7.4%
**Israel**	135	17.9%	55.9%	17.9%
**Italy**	1007	8%	71.8%	8%
**Latvia**	1192	15.6%	62.7%	15.6%
**Lebanon**	132	12%	72.8%	12%
**Moldova**	213	21.7%	45.8%	21.7%
**Norway**	1063	9.9%	76.1%	9.9%
**Peru**	208	16.7%	54.9%	16.7%
**Portugal**	262	20.6%	58.2%	20.6%
**Qatar**	105	10.5%	75.2%	14.3%
**Romania**	188	8.7%	71.1%	8.7%
**Slovenia**	260	5.9%	82.1%	5.9%
**Switzerland**	170	12.8%	70.6%	12.8%
**Turkey**	214	17.5%	60.4%	17.5%
**United Arab Emirates**	130	11.5%	76.2%	12.3%
**United Kingdom**	696	17.5%	66.5%	16%
**United States**	373	18.3%	67.8%	13.9%
**Total**	**10,293**			

**Fig 7:**
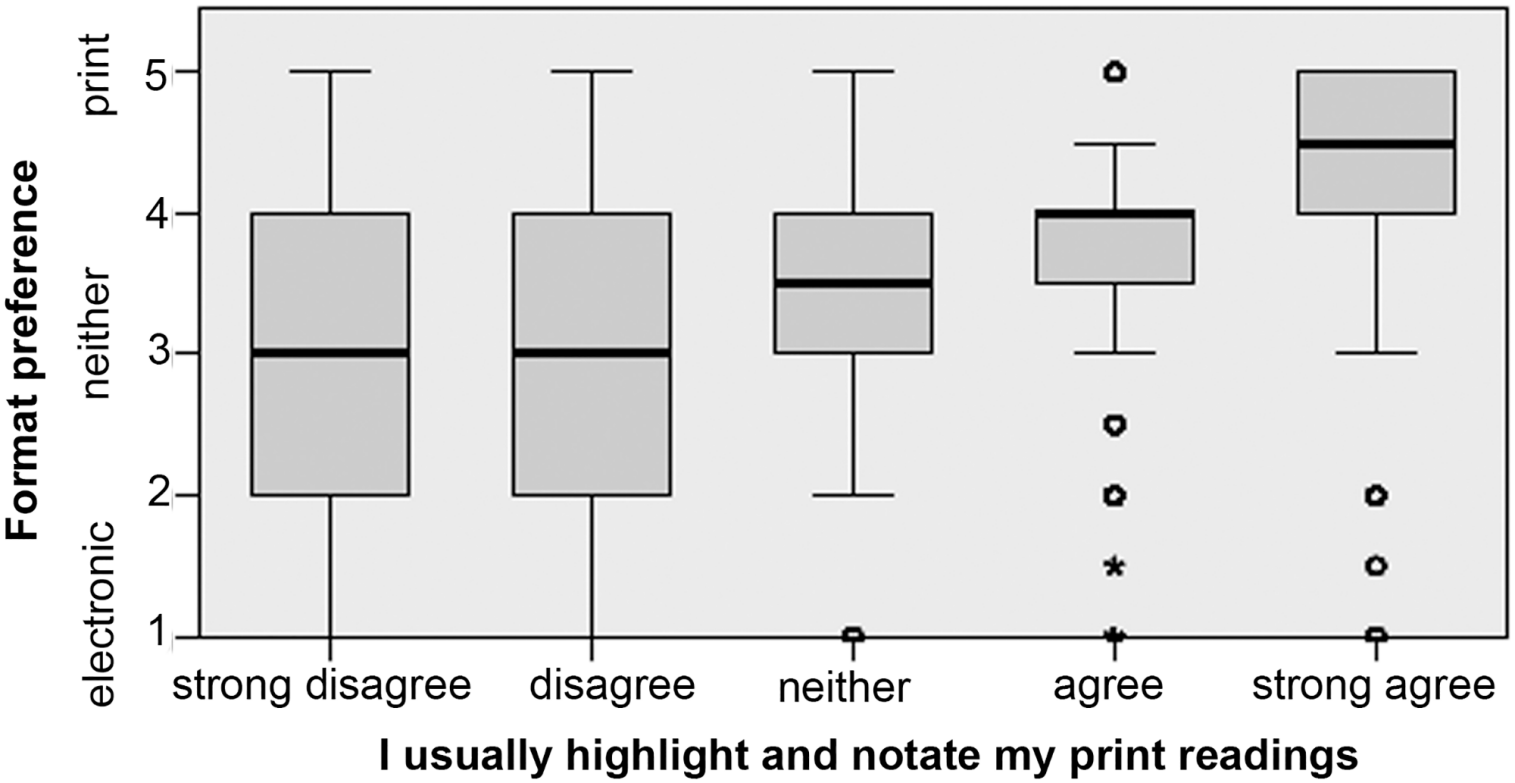
Responses to the statements “I usually highlight and notate my printed course readings” plotted against overall format preference. Relationship is significant with a small effect size (Kruskal-Wallis H = 2,362.222, p < .0005, n = 10,293, η^2^ = 0.23).

**Fig 8:**
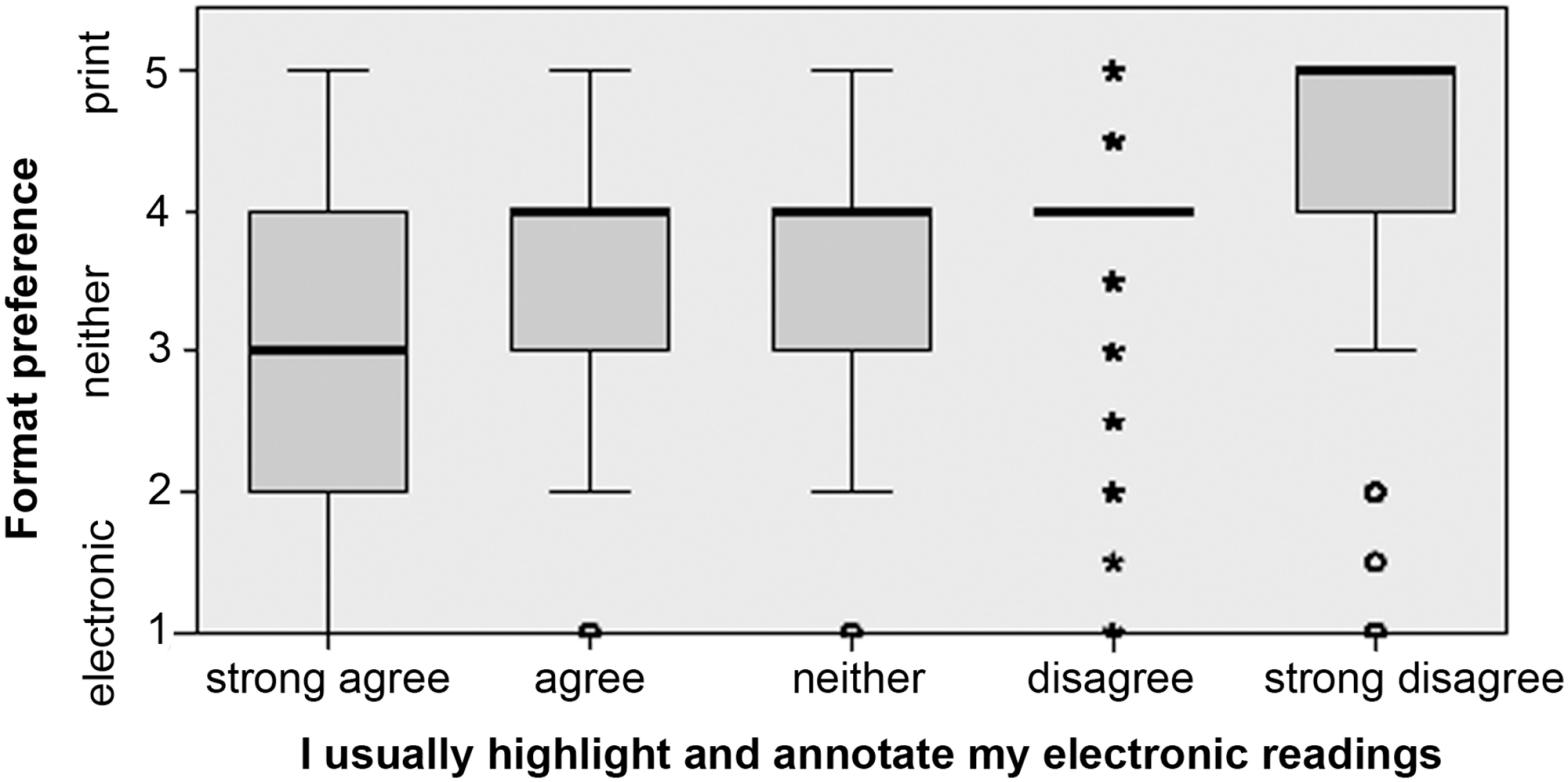
Responses to the statement “I usually highlight and annotate my electronic course readings” plotted against overall format preference. Relationship is significant with a small effect size (Kruskal-Wallis H = 2,067.093, p < .0005, n = 10,293, η^2^ = 0.20).

Highlighting and annotating important texts are common learning strategies that demonstrate an effort to engage with a reading for effective comprehension and retention. Among our respondents, 83.6% agreed or strongly agreed that they usually highlight and annotate their printed course readings, but only 24.11% said they did the same with electronic readings. In each case, format preference is correlated to a small degree with the use of text engagement tools in that format. Figs 7 and 8 show the correlation between self-reported highlighting and/or note-taking behavior and expressed format preference.

#### R3. Does the length of reading influence format preference?

**Fig 9:**
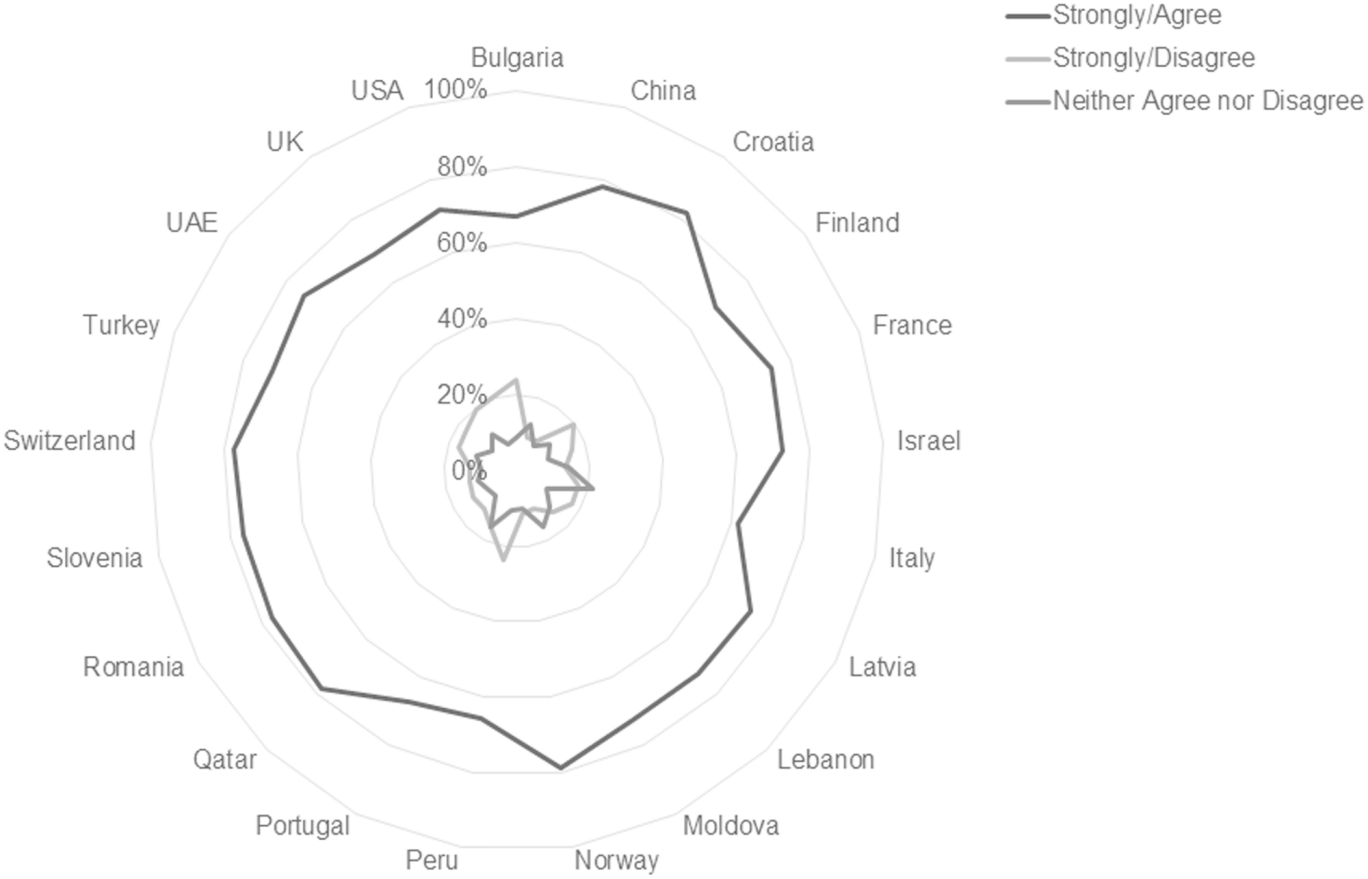
Responses to the statement “If an assigned reading is 7 pages or more, I prefer to read it in print” reported by country. [n = 10293; Agree/Strongly Agree n = 7496, 72.83%; Neither Agree nor Disagree n = 1197, 11.63%; Disagree/Strongly Disagree n = 1600, 15.54%].

**Fig 10:**
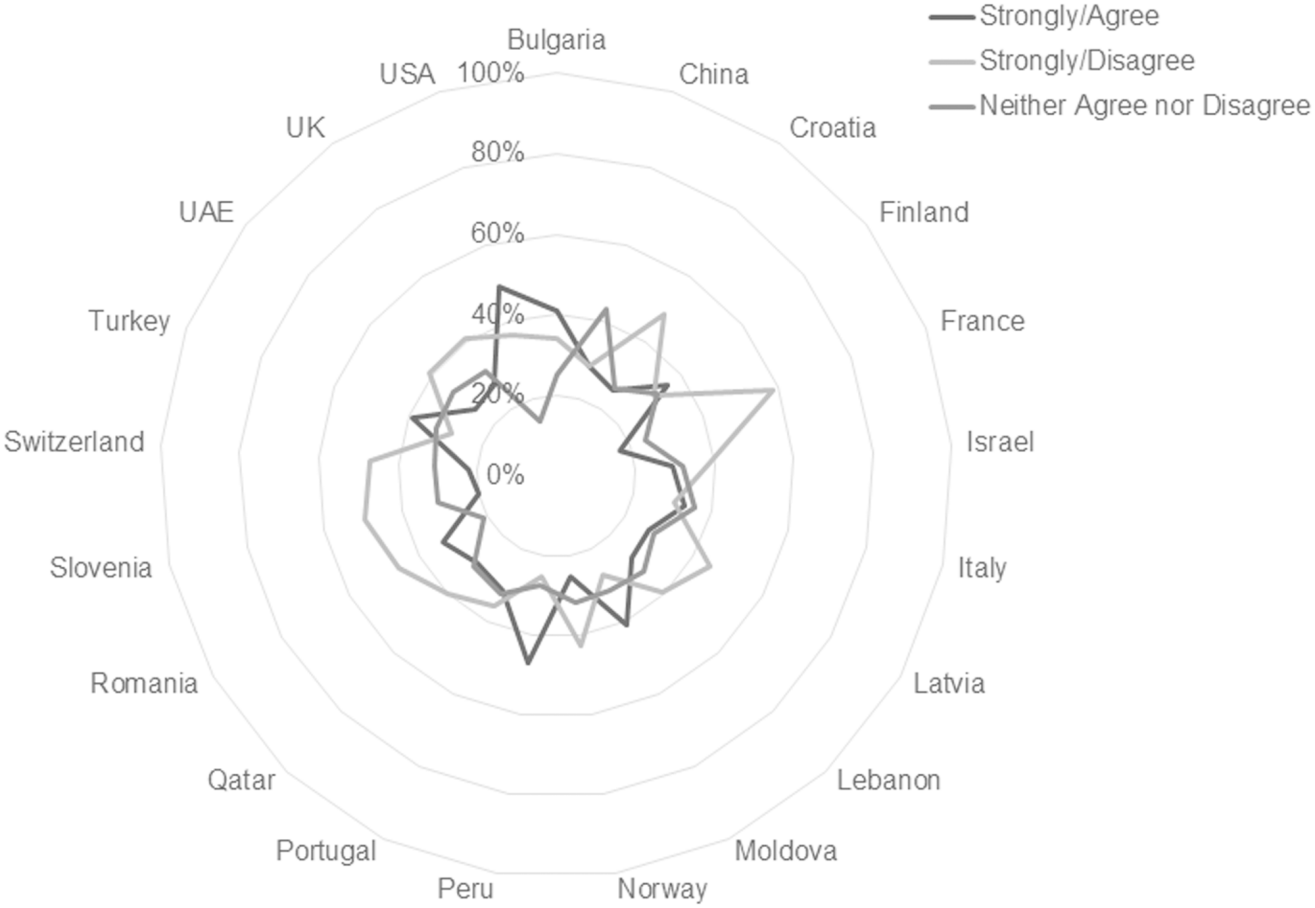
Responses to the statement “If an assigned reading is less than 7 pages, I prefer to read it electronically” reported by country. [n = 10293; Agree/Strongly Agree n = 2960, 28.76%; Neither Agree nor Disagree n = 3160, 30.70%; Disagree/Strongly Disagree n = 4173, 40.54%].

Earlier studies have observed that format preference can depend on the length of the reading, in that print can be preferred for longer readings while electronic is adequate or preferable for shorter material [8,25]. Mizrachi [25] attempted to define what constitutes a longer or shorter reading by comparing participant preferences across three reading length categories: readings under five pages, readings 5–10 pages, and material over ten pages long. No differences were found between responses to the latter length categories. ARFIS thus asks about format preferences for just two categories: reading material of seven pages or more in length, and material less than seven pages. As illustrated in Fig 9 and Table 8, participants demonstrate consensus in their preference for print format for longer readings (72.83%). Results for shorter readings still show that print is preferred, but there are many variations by country as shown in Fig 10 and Table 9. Overall, 30.7% answered they neither agreed nor disagreed.

**Table 8:** Responses to the statement “If an assigned reading is 7 pages or more, I prefer to read it in print” reported by country.

Country	n	% Agree/Strongly Agree	% Disagree /Strongly Disagree	% Neither Agree nor Disagree
**Bulgaria**	237	67.3%	23.5%	9.2%
**China**	1165	78.3%	9%	12.6%
**Croatia**	232	82.2%	9.5%	8.2%
**Finland**	681	69.2%	19.4%	11.4%
**France**	1630	75.4%	16.1%	9.4%
**Israel**	135	72.4%	13.4%	14.2%
**Italy**	1007	60.7%	17.8%	21.5%
**Latvia**	1192	73.4%	17.2%	9.4%
**Lebanon**	132	72%	14.4%	13.6%
**Moldova**	213	72.7%	10.9%	16.5%
**Norway**	1063	79.2%	11.5%	9.3%
**Peru**	208	67.2%	22.5%	10.3%
**Portugal**	262	67%	16.5%	16.5%
**Qatar**	105	78.1%	13.3%	8.6%
**Romania**	188	77.2%	13.5%	9.2%
**Slovenia**	260	75.8%	13.3%	10.9%
**Switzerland**	170	78.2%	11.6%	10.3%
**Turkey**	214	71.1%	17%	11.3%
**United Arab Emirates**	130	73.9%	17.7%	8.5%
**United Kingdom**	696	69%	19.4%	12.6%
**United States**	373	72.2%	20.5%	7.4%
**Total**	**10,293**			

**Table 9:** Responses by country to the statement “If an assigned reading is less than 7 pages, I prefer to read it electronically”.

Country	n	% Agree/Strongly Agree	% Disagree /Strongly Disagree	% Neither Agree nor Disagree
**Bulgaria**	237	40.7%	34%	25.2%
**China**	1165	28.5%	28.4%	43.1%
**Croatia**	232	25.1%	48.5%	26.4%
**Finland**	681	35.8%	31.2%	33%
**France**	1630	17.1%	57.9%	25%
**Israel**	135	29.9%	38.1%	32.1%
**Italy**	1007	32.7%	30.6%	36.6%
**Latvia**	1192	26.7%	45%	28.2%
**Lebanon**	132	28%	39.2%	32.8%
**Moldova**	213	41.5%	26.9%	31.6%
**Norway**	1063	25.3%	32.6%	32.2%
**Peru**	208	46.1%	25.5%	28.4%
**Portugal**	262	31.8%	36%	32.2%
**Qatar**	105	29.5%	40%	30.5%
**Romania**	188	32.6%	46.2%	21.2%
**Slovenia**	260	20.3%	49.6%	30.1%
**Switzerland**	170	23.1%	46.2%	30.8%
**Turkey**	214	38.7%	28.8%	32.5%
**United Arab Emirates**	130	26.2%	40.8%	33.1%
**United Kingdom**	696	27.2%	41.3%	30.5%
**United States**	373	49.2%	36.9%	13.9%
**Total**	**10,293**			

**Fig 11:**
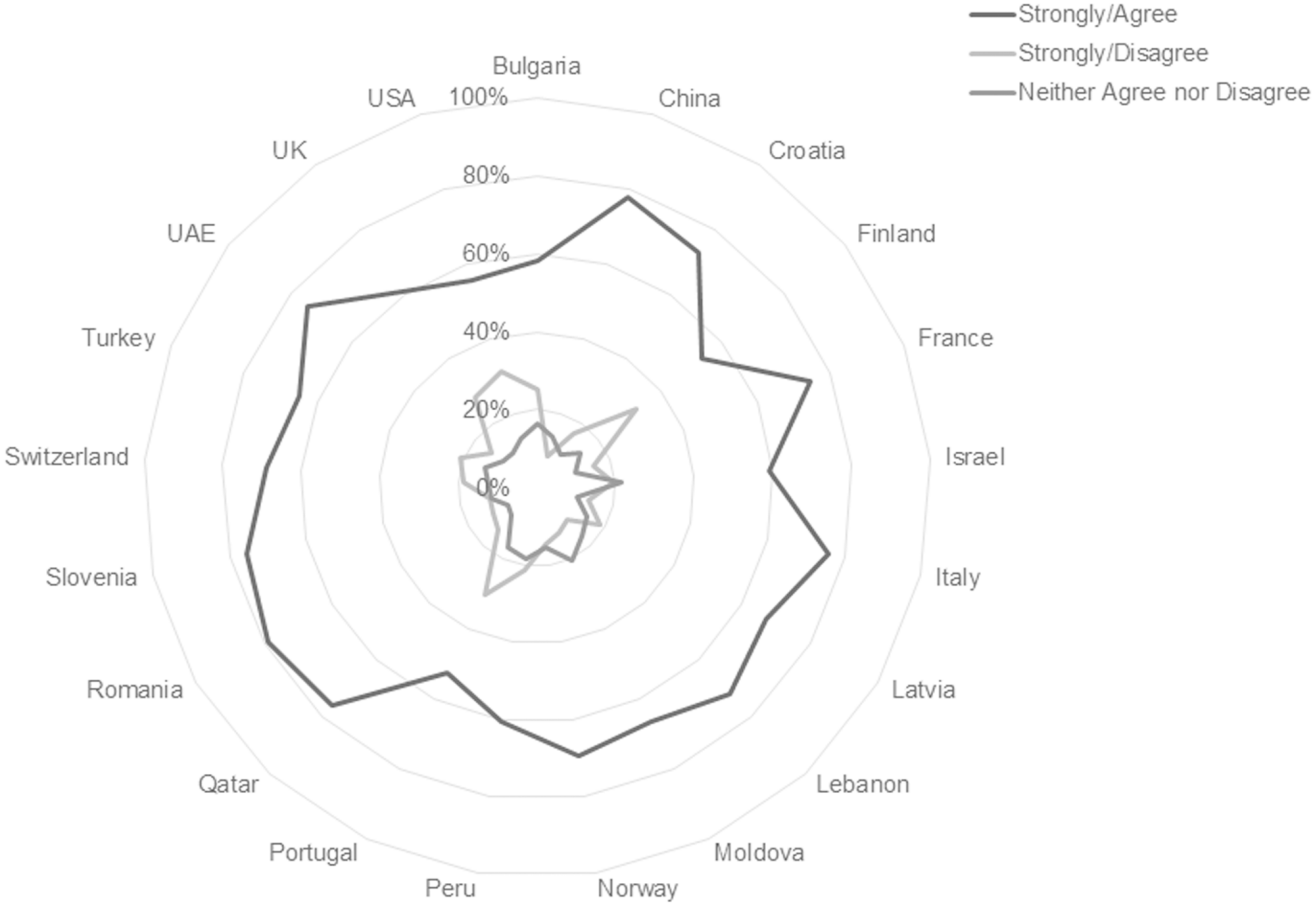
Responses to the statement “I prefer to print out my course readings rather than read them electronically” reported by country. [n = 10293; Agree/Strongly Agree n = 7087, 68.85%; Neither Agree nor Disagree n = 1354, 13.15%; Disagree/Strongly Disagree n = 1852, 17.99%].

Printing out electronic material to read or review involves more effort, time and expense than reading online, yet 68.85% of the students agreed or strongly agreed that they prefer to do so. The consistency of these responses is illustrated in Fig 11 and Table 10, as well as some variations among countries. Responses to this question were highly consistent across academic rank, ranging from a low of 67.6% agreement to a high of 70.6% agreement spanning first-year to postgraduate students.

**Table 10:** Responses to the statement “I prefer to print out my course readings rather than read them electronically” reported by country.

Country	n	% Agree/Strongly Agree	% Disagree /Strongly Disagree	% Neither Agree nor Disagree
**Bulgaria**	237	58.4%	24.7%	16.8%
**China**	1165	78%	8.4%	13.6%
**Croatia**	232	73%	16.5%	10.4%
**Finland**	681	53.3%	32.6%	14.1%
**France**	1630	74.6%	15.5%	9.9%
**Israel**	135	59%	19.4%	21.6%
**Italy**	1007	76%	14.4%	9.6%
**Latvia**	1192	67.1%	18.4%	14.4%
**Lebanon**	132	70.4%	12%	17.6%
**Moldova**	213	66.5%	12.7%	20.8%
**Norway**	1063	69.6%	14.6%	15.8%
**Peru**	208	61.3%	20.1%	18.6%
**Portugal**	262	52.9%	30.6%	16.5%
**Qatar**	105	76.2%	14.3%	9.5%
**Romania**	188	79.3%	13%	7.6%
**Slovenia**	260	75.8%	12.5%	11.7%
**Switzerland**	170	70.5%	17.9%	11.5%
**Turkey**	214	65.1%	21.2%	13.7%
**United Arab Emirates**	130	74.6%	14.6%	10.8%
**United Kingdom**	696	61.1%	27.9%	11%
**United States**	373	55.5%	31.4%	13.1%
**Total**	**10,293**			

#### R4. Is the language of the reading a confounding factor in evaluating format preference?

Many students worldwide must read material for their coursework which is in neither their native language nor the language of instruction at their institution, a potentially confounding factor in evaluating reading preference. Three statements in this survey sought to ascertain whether participants would be influenced in their responses based on linguistic considerations. Responses to two of them: *I prefer to read course readings which are in my native language electronically rather than print; I prefer reading foreign language material in print than electronic*, indicate that students prefer print for their native language, but this may just be a reflection of their overall print preference. In terms of item reliability, these two statements did not correlate well with each other or with the rest of the scale, which limits the extent to which one can draw conclusions from this data. One statement spoke directly to the impact of the language on format preference: *My preferred reading format*, *electronic or print*, *depends on the language of the reading*. Just over 15% of respondents agreed to any extent with this statement; 56.11% of students worldwide disagreed or strongly disagreed with this statement; while a further 28.04% neither agreed nor disagreed. This indicates that most participants do not believe that the language of presentation influences their format preferences, but that a minority do.

#### Characteristics of participants preferring e-formats

A minority of respondents expressed a preference for e-reading for academic materials. Out of the 10,293 respondents, 1033 (10.04%) had a median scale score of 2.5 or less, indicating a preference for electronic reading, where a score of 3 indicates a neutral response with an average ‘neither agree nor disagree’ to preference statements. In investigating the characteristics of respondents preferring e-formats, we considered the 805 (7.8%) with a median scale score of 2 or less, putting them firmly out of neutral territory, wherein both print and digital formats might seem equally appealing, and into a group of respondents whose average responses indicate a clear preference for electronic reading over print. Of this subgroup, 52.2% are male (versus 28.9% of the total sample), 32.9% are graduate-level students (versus 27.5% of the total sample), and 5.8% reported having visual limitations (versus 16.4% of the total sample). Table 11 lists the countries represented by e-preferring respondents and their proportions.

**Table 11:** Country origin of e-preferrers and all respondents, n = 805, 10,293.

Country	Total n	Total %	E-preferring %	E-preferring n
**Bulgaria**	237	2.30	4.8	39
**China**	1165	11.32	3.4	27
**Croatia**	232	2.25	1.4	11
**Finland**	681	6.62	9.6	77
**France**	1630	15.84	10.4	84
**Israel**	135	1.31	1.1	9
**Italy**	1007	9.78	5.5	44
**Latvia**	1192	11.58	15.2	122
**Lebanon**	132	1.28	1.2	10
**Moldova**	213	2.07	1.1	9
**Norway**	1063	10.33	10.3	83
**Peru**	208	2.02	3.6	29
**Portugal**	262	2.55	4.1	33
**Qatar**	105	1.02	1.0	8
**Romania**	188	1.83	1.4	11
**Slovenia**	260	2.53	1.6	13
**Switzerland**	170	1.65	2.0	16
**Turkey**	214	2.08	3.1	25
**United Arab Emirates**	130	1.26	1.4	11
**United Kingdom**	696	6.76	10.7	86
**United States**	373	3.62	7.2	58
**Total**	**10,293**	**100**	**100**	**805**

Device usage patterns across this group exhibit some differences from the full dataset. Table 12 shows how reported device usage among this group compares with both the full response set and a mirrored subset of print-preferrers with scale scores of 4.0–5.0. The most popular devices in every group are laptops, with phones in a distant second place. However, those with a clear preference for e-text report more frequent usage of every type of device for e-reading, and are twice as likely as strong print-preferrers to report using a tablet to read electronically. They are more than twice as likely to report using a dedicated e-reader, and almost four times as likely to report using audio reading.

**Table 12:** Device usage by format preference.

I read my e-readings on a…	Strong e-preference (n = 805)	All (n = 10,293)	Strong print preference (n = 7489)
**Laptop**	86.8%	80.9%	80.2%
**Desktop**	42%	30.54%	28.1%
**iPad/Tablet**	48%	28.43%	24.2%
**E-reader**	12.3%	7.00%	5.4%
**Phone**	49.2%	36.83%	33.3%
**Audio**	5.1%	1.97%	1.3%
**Never/none**	0.2%	4.34%	5.8%

### Exploratory findings

Several post-hoc findings from this dataset are of interest. While majorities of respondents across academic levels indicated an overall preference for print material, the intensity of this preference weakens slightly across the dataset as participants rise in academic rank. These groups appeared significantly unique in data subset A, (n = 5145, z = 41.916, η^2^ = .003, p < .001), significantly unique in data subset B, (n = 5148, z = 38.105, η^2^ = .003, p < .001) and mean ranks for the full dataset are shown in Table 13 (n = 10,293, z = 76.858, η^2^ = .007, p < .001). The effect size estimate for these differences is very small, but the ranks are linear, with smaller majorities of students preferring print format with each rise in academic rank.

**Table 13:** Homogenous subsets by year level, n = 10,293.

		Subset		
		1	2	3
**Sample**^a^	**Doctoral** (n = 584)	4652		
	**Master** (n = 2253)	4857		
	**Fourth-year** (n = 1237)	5040	5040	
	**Third-year** (n = 1859)		5200	5200
	**Second-year** (n = 1949)		5262	5262
	**First-year** (n = 2074)			5436
	**Other** (n = 337)			5597
**Test Statistic**		7.803	4.737	10.798
**Sig. (2-sided test)**		0.02	0.094	0.013
**Adjusted Sig. (2-sided test)**		0.047	0.205	0.022

^a^ Subsets are based on asymptotic significances. The significance level is .01. Higher mean ranks indicate greater intensity of print preference.

24.9% of the 584 doctoral-level respondents in this dataset are sourced from Norway, 18% from China and 13.7% from Finland. Of the master-level respondents, 16.2% are sourced from Latvia, 16% are sourced from China, and 12.7% are from Norway. As a percentage of total respondents from each country, Israel has the highest proportion of graduate students responding, with 54.8% at the master or doctoral level, followed by Turkey at 43.4%, Peru at 41.3%, Norway at 40.5% and China at 40%.

Academic rank tends to be associated with age, and for this reason we also present analysis drawn from the full dataset related to age in Table 14. The intensity of print preferences based on the full scale does vary in a statistically significant manner by age (n = 10,083, z = 88.004, η2 = .011, p < .001). For the purposes of granularity, respondent ages have been grouped by single years from 18–35, which accounts for 90.8% of respondents at n = 9,356. Those aged 36 and over are presented collectively and this accounts for 727 or approximately 7% of the total sample. While these groups are statistically significantly different with a very small effect size, there is not a strong linearity to the mean ranks by age. 18 and 19 year olds show slightly higher intensity for print preference than the rest of the respondents, yet their response patterns are also statistically indistinguishable from 28 year olds. As with academic ranks, majorities of respondents across age groups prefer print formats.

**Table 14:** Homogenous subsets by age, n = 10,293.

		Subset				
		1	2	3	4	5
**Sample age**^1^	**34**	4512				
	**32**	4642				
	**33**	4643				
	**30**	4702				
	**36–65**	4823				
	**31**	4846				
	**29**	4847				
	**25**	4861				
	**26**	4869	4869			
	**35**	4874	4874	4874		
	**27**	4953	4953	4953		
	**23**	5061	5061	5061		
	**24**	5113	5113	5113	5113	
	**21**	5162	5162	5162	5162	
	**28**	5236	5236	5236	5236	5236
	**22**	5246	5246	5246	5246	5246
	**20**		5364	5364	5364	5364
	**19**			5429	5429	5429
	**18**					5763
	**Other**^2^			5714	5714	
**Test Statistic**		30.139	16.917	17.638	14.039	13.861
**Sig. (2-sided test)**		.011	.031	.024	.029	.017
**Adjusted Sig. (2-sided test)**		.014	.068	.053	.081	.054

^1^ Subsets are based on asymptotic significances. The significance level is .01. Higher mean ranks indicate greater intensity of print preference.

^2^ Missing/unknown age data, n = 210

## Discussion

### Gender

The overall study sample is skewed female with nearly 70% of the respondents being female and approximately 4.6% of the scale score variability is attributable to gender. A higher female response rate is not unusual in survey research [35]. While male and female respondents prefer print at a similar rate, the difference shows up in the intensity of print preference, with female respondents being slightly more likely to use the extreme ends of the Likert-type scale on preference questions. This is possibly a result of female survey-response patterns more than a meaningful difference in format preferences. The size and nature of the effect leads us to conclude that the gender imbalance in the participant sample does not meaningfully influence the overall data trends and conclusions. That said, we also note that among the small percentage of respondents who report a preference for digital formats, males are overrepresented, making up over half of this group while being less than a third of the total sample.

### Country

The small effects on overall full-scale response distribution by country point to a remarkably consistent international participant sample. We had hypothesized that educational style, levels of socioeconomic development, and/or technological readiness associated with geopolitical boundaries might influence the format preferences of university learners. However, the data gathered cannot support these hypotheses. The data gathered points to statistically significant but very small differences in responses across countries, and some of the variations that occur in any large, cross-national study must be attributed to documented cultural variation in survey response patterns [36,37]. We feel confident based on this dataset that these factors are less influential to student format preferences and experiences than the university environment, learner characteristics, reading tasks and format characteristics documented across the existing literature. This finding is also significant in that it provides evidence that university student populations sampled in the United States or other centers for research data production are generalizable to a global student population. However, some data points stand out and are worth considering in greater depth.

First, in terms of device usage, we pointed out that respondents in China reported using a broader range of types of electronic devices for e-reading than their peers across the cohort, and suggested that this may reflect something about China’s educational culture and/or access to technology among university students. At the same time, respondents from China prefer print formats in majorities that are statistically comparable to the rest of the respondents. The patterns of device usage reported by participants are not obviously linked to the format preferences gathered, though additional statistical analysis may yield further insight.

It is notable that nearly 10% of the 1630 respondents from France say they do not read electronically at all, constituting over 1/3 of all respondents who said they do not read electronically across the full dataset. This rate is closely followed by Lebanon, Qatar, and the UAE, whose respondents say they do not read electronically at rates of 9.8%, 8.6%, and 7.7% respectively, all well above the global average rate of 4.3%. France is known for having a fairly robust book culture, with a thriving independent book industry and a high rate of annual book consumption among readers, which might be an explanatory factor [38]. However, this is not the case with Qatar. What do these nations have in common? It is possible that cost and access issues frame non-electronic reading in these places. For instance, France’s 1981 “Lang Law” fixes the prices of books sold in France and prohibits discounting, even for online retailers, and this policy was extended to digital books [39]. As such, the economic advantages which drive electronic usage in other places may be neutralized to a degree in France, for both individuals and institutions. The other three countries are the only Arab-league countries in this dataset. We would note that the Arab world at large has been slow to adopt and support general e-commerce activities compared to the rest of the world, mainly due to regulatory and consumer trust issues [40]. It is conceivable that geographic embargoes from content distributors, online purchasing restrictions from Arab banks and other regional factors could help explain the elevated levels of non-ereadership across these countries. Progress on resolving these barriers is being made in many Arab countries and it would be worthwhile to explore how e-readership patterns develop here over time.

Finally, Fig 11 above illustrates data on respondents who prefer to print out course readings rather than read them electronically. Majorities of respondents worldwide agree that they prefer to do this, but there is some variability. This is a behavior that could be influenced by environmental concerns with paper printing, and/or cost concerns, depending on access to and charges for printing services at the respondents’ homes or academic institutions.

### Academic rank

While the size of the effect of differences in responses by academic rank is statistically small, its existence and the linearity of the relationship is unlikely to be due to chance and this is of interest and merits further investigation. Respondents of a higher academic rank show slightly lower rates of print preference. This could be explained in part by the types of reading materials and tasks with which higher-level students are engaging. Upper-undergraduate and graduate students are more likely to rely on academic articles and scholarly literature than textbooks, and these sources tend to be shorter and perhaps more retrievable or accessible electronically than textbooks. As noted in the results, students at all academic levels reported a preference for print when reading texts of 7 pages or longer at statistically similar rates ranging from 67.6–70.6% for each year group. On this item there is no linear relationship to academic rank.

From a usability perspective, the slight tendency away from print preference among higher academic ranks could also reflect an accumulation of experience with e-formats and/or academic reading tasks that make the utilization of e-formats comparatively easier for these students.

The relationship between academic rank and format preference does not correspond directly to aging in general. The youngest students, aged 18 and 19, exhibit slightly more intense print preference than the rest of the group, but beyond this there appears to be little linearity to the relationship between age and reported format preferences.

### Learning engagement

The relationship between using the text engagement tools of a format, such as highlighting and notetaking, and the general preference for that format, is clear but lacks directionality. From this dataset, we cannot know whether, for example, students prefer e-texts in part because they are already comfortable with e-reading platform tools like digital highlighting and notetaking, or whether they have come to utilize those tools often because they first preferred e-reading. The same can be said for those who prefer print, and use physical highlighters and pens for notation.

### Theoretical directions

A majority of respondents reported perceiving that they remember information better and focus better on information presented in print format, regardless of the types of devices used for e-reading. However, e-preferring readers more frequently report employing a range of devices for e-reading, including tablets, dedicated e-readers and phones. This data may lend credence to the usability [16] and mental mapping [23] perspectives on learning from e-text, both of which leave an opening for the features of hardware and software for e-text presentation to be designed and innovated to support cognitive learning processes rather than detract from them. However, the directionality of the data presented here remains unclear, and the range of presentations and hardware types available on the market today in any one of these device subcategories is growing. More granular data gathering is indicated to more clearly establish both directionality and specific device features that may correlate with higher electronic reading preference.

The results indicate that a perception of superior information retention and focus with print formats correlates to a preference for use of that format. While factors other than learning performance clearly play a role in determining preference, this data suggests that readers have a sense of awareness about the impacts of format on their own learning and that this factor does contribute to format decisions and behavior. In using theoretical perspectives to evaluate format choices and preferences, this data would suggest that ‘cognitive effort’ or effort to learn—even if it is only *perceived* effort—would influence behavior and should be included in judgments of overall effort expended on the utilization of text for a given purpose.

### Implications

The practical implications from this data inform tertiary educators and educational institutions worldwide. There is no doubt that the affordability, accessibility, and searchability of e-formats will continue to benefit learners and educators in important ways. While limitations and disadvantages of e-formats are captured in the data presented here, this is not a reason to stop using these formats or revert to only text-based collections and course materials. Rather, this data presents opportunities to balance more carefully the competing concerns about formats, learning, costs and access. It also presents opportunities for technologists, developers and instructional designers to look for ways of improving the usability and likeability of e-formats for tertiary learners.

Small changes to instructional design within tertiary classrooms may be both feasible in the near-term and beneficial for learners. In environments where we often employ a mix of hard copy and digital resources, the finding that e-formats are more acceptable for shorter texts than longer ones implies that educators may want to choose digital formats for shorter readings and ensure that print copies are available in one way or another for longer readings.

The findings that large majorities of learners believe they learn better from and focus better on materials in print implies that the provision of print materials for learning remains a desirable option. However, many tertiary institutions charge for printing services, and these policies might be worth reviewing for equity purposes. Students who find it beneficial to learn from print texts but must pay a premium to print out course materials may be at an economic disadvantage compared to their peers, and this is an institution or department-level facet of the instructional environment that can be considered in light of these findings.

The finding that students who utilize learning engagement features of e-texts, such as highlighting and annotation tools, are more likely to be neutral about or prefer e-formats, suggests the possibility that greater facility with these functions could influence preferences towards electronic. Instructional designers could work towards helping students acquire the prerequisite knowledge to leverage digital texts through more explicit instruction on the navigation of e-formats. More research is necessary to test this hypothesis, however, because the data presented here indicates only that a relationship between learning engagement behaviors and preference exists, and does not indicate a direction for that relationship.

In the longer term, user interface and hardware designers can use preference and behavioral data such as that presented here to steer the development of learner-friendly platforms and devices.

## Conclusions, limitations, future directions

The findings of this study show that majorities of the university students surveyed worldwide self-report that they prefer to read their academic materials in print format, and believe that they learn better and focus better on material presented in print. This perception among students is consistent with prior comprehension studies showing that print format is better for granular recollection of information and in-depth understanding. Statistically significant differences in the intensity of that preference across countries internationally are present but very small. In a large cross-national survey study, some variation is expected on the basis of culturally-linked survey response patterns.

Respondents are more likely to prefer print formats for lengthier readings, and are more likely to prefer a format when they report more learning engagement behaviors, such as highlighting and notetaking, in that format. Post-hoc analysis shows a small but regular relationship between format preferences and academic rank, with higher-ranking respondents exhibiting less strong preference for print formats.

Implications for educators and tertiary institutions center on instructional design and format choices for course materials, as well as the provision of printing services and reserves services to augment e-course materials for students who may educationally benefit from such services. Implications for researchers are that findings about user format preferences from studies around the world are likely generalizable to similar contexts in other countries.

The study asked participants about their preferences, but also about their behaviors and learning. Self-reports are a good measure of personal perspectives and preferences, but are not always consistent with actual behaviors. Prior research has found that students are able to predict or judge their learning performance to varying degrees of accuracy depending on the circumstances. Therefore, the data reported in this study pertaining specifically to behavior and learning/focus have to be considered alongside other empirical performance data concerning print and digital reading, comprehension and learning. Evidence of this nature available to date suggests that university students often do perform better on reading comprehension tests, particularly regarding recall of granular information, when reading from print [4,12]. The beliefs expressed by the majority of participants in this study concerning their own memory and focus with print materials appears consistent with other performance-based studies, and indicates that some learners may be able to accurately perceive the effect that format/medium has on their learning and retention, and integrate this knowledge into their overall preference. However, it is apparent that considerations beyond comprehension factor into reader format preferences and that in many cases, comprehension performance and format preferences may not align. The conclusions that can be drawn about the nature of the relationship between comprehension and format preference are tentative and require further study.

This study has also dealt exclusively with the perceptions of university students about reading academic course materials. This population specifically makes use of academic course materials in order to learn, apply this learning in an academic settings and earn academic credit. The nature of the task or purpose for reading is very likely to influence user preference for format, and this makes it important not to attempt to extend the findings of this study to other types of reading for other purposes, including leisure reading, news reading, workplace-based reading or other contexts. Examples from prior literature illustrate that readers may prefer electronic texts for non-academic sorts of reading tasks, tasks such as leisure reading or reading for review [12].

This paper presents a general picture of current international trends, but more statistical analysis of the data and continued explanatory research is needed to increase the depth of our understanding of this phenomenon and to monitor trends. In particular, additional research is needed to identify the conditions and factors that coincide with the minority preference for electronic material, as this may inform the development and delivery of e-texts under optimal conditions for usability and learning. Moreover, while this data conclusively illustrates that a majority of tertiary students worldwide prefer print formats, there may be a variety of reasons contributing to this outcome. A pending analysis of the qualitative data gathered as part of this project may yield insights about respondents who prefer e-reading for academic texts and the perceptions and more explanatory data on the factors driving preferences for these learners. Research and practice in education, psychology, information science and human-computer interaction are informed by this data and its implications.

The purpose and nature of a reading task, whether academic or otherwise; the length of a reading; characteristics of the environment, such as costs and convenience; and to some extent the characteristics of a reader, such as academic seniority or presence of visual limitations; all interact together to influence a user’s preference for reading format. The manner in which the relationship between reading task, socio-environmental factors and reader characteristics combine to influence format preferences and behavior is important to understand for educators attempting to make instructional design decisions as well as technologists and publishers interested in both steering and responding to consumer reading habits and preferences. More empirical data and theoretical work in this area is necessary to clarify how learner preferences and behaviors can be influenced or catered to through design, presentation, and hardware choices.

## Supporting information

S1 Data ARFIS 1 dataset. (CSV)

## References

[1] Lenhart A. Teens, Social Media & Technology Overview 2015. Washington, D.C.; 2015.

[2] Houghton S, Hunter SC, Rosenberg M, Wood L, Zadow C, Martin K, et al Virtually impossible: limiting Australian children and adolescents daily screen based media use. BMC Public Health. BioMed Central; 2015;15: 5 doi: 10.1186/1471-2458-15-5 25613954 10.1186/1471-2458-15-5 PMC4324783

[3] Rideout VJ, Ulla MA, Foehr G, Roberts DF. Generation M2: Media in the Lives of 8-to 18-Year-Olds. Menlo Park, CA; 2010.

[4] Singer LM, Alexander PA. Reading on paper and digitally: What the past decades of empirical research reveal. Rev Educ Res. 2017; 34654317722961. doi: 10.3102/0034654317722961

[5] Anderson R, Pearson PD. A Schema-Theoretic View of Basic Processes in Reading Comprehension Handbook of reading research. 1984 pp. 255–291. doi: 10.1017/CBO9781139524513.007

[6] Dilevko J, Gottlieb L. Print sources in an electronic age: a vital part of the research process for undergraduate students. J Acad Librariansh. 2002;28: 381–392. doi: 10.1016/S0099-1333(02)00341-5

[7] Liu Z. Print vs. electronic resources: A study of user perceptions, preferences, and use. Inf Process Manag. 2006;42: 583–592. doi: 10.1016/j.ipm.2004.12.002

[8] Li C, Poe F, Potter M, Quigley B, Wilson J. UC Libraries Academic e-Book Usage Survey. 2011.

[9] Foasberg NM. Student reading practices in print and electronic media. Coll Res Libr. 2014;75: 705–723. doi: 10.5860/crl.75.5.705

[10] Baron N. Words onscreen: The fate of reading in a digital world. Oxford, New York: Oxford University Press; 2015.

[11] Mizrachi D, Boustany J, Kurbanoğlu S, Doğan G, Todorova T, Vilar P. The Academic Reading Format International Study (ARFIS): Investigating Students Around the World In: Kurbanoğlu S, Boustany J, Špiranec S, Grassian E, Mizrachi D, Roy L, et al, editors. Communications in Computer and Information Science. Cham: Springer International Publishing; 2016 pp. 215–227.

[12] Singer LM, Alexander PA. Reading Across Mediums: Effects of Reading Digital and Print Texts on Comprehension and Calibration. J Exp Educ. 2017;85: 155–172. doi: 10.1080/00220973.2016.1143794

[13] Wang S, Bai X. University Students Awareness, Usage and Attitude Towards E-books: Experience from China. J Acad Librariansh. 2016;42: 247–258. doi: 10.1016/j.acalib.2016.01.001

[14] Bansal G. Continuing e-book use: role of environmental consciousness, personality and past usage. J Comput Inf Syst. 2011;52: 93–104.

[15] Revelle A, Messner K, Shrimplin A, Hurst S. Book Lovers, Technophiles, Pragmatists, and Printers: The Social and Demographic Structure of User Attitudes toward e-Books. Coll Res Libr. 2012;73: 420–429. doi: 10.5860/crl-288

[16] Wästlund E. Experimental studies of human-computer interaction: working memory and mental workload in complex cognition. Department of Psychology, Gothenburg University 2007.

[17] Stoop J, Kreutzer P, Kircz J. Reading and learning from screens versus print: a study in changing habits: {Part} 1 –reading long information rich texts. New Libr World. 2013;114: 284–300. doi: 10.1108/NLW-01-2013-0012

[18] Eshet-Alkalai Y, Geri N. Does the medium affect the message? {The} influence of text representation format on critical thinking. Hum Syst Manag. 2007;26: 269–279.

[19] Ackerman R, Goldsmith M. Metacognitive regulation of text learning: On screen versus on paper. J Exp Psychol Appl. 2011;17: 18–32. doi: 10.1037/a0022086 21443378 10.1037/a0022086

[20] Mangen A, Walgermo BR, Brønnick K. Reading linear texts on paper versus computer screen: Effects on reading comprehension. Int J Educ Res. 2013;58: 61–68. doi: 10.1016/j.ijer.2012.12.002

[21] Subrahmanyam K, Michikyan M, Clemmons C, Carrillo R, Uhls YT, Greenfield PM. Learning from paper, learning from screens: Impact of screen reading and multitasking conditions on reading and writing among college students. Int J Cyber Behav Psychol Learn. 2013;3: 1–27. doi: 10.4018/ijcbpl.2013100101

[22] Sidi Y, Ophir Y, Ackerman R. Generalizing screen inferiority—does the medium, screen versus paper, affect performance even with brief tasks? Metacognition Learn. 2016;11: 15–33. doi: 10.1007/s11409-015-9150-6

[23] Hou J, Rashid J, Lee KM. Cognitive map or medium materiality? Reading on paper and screen. Comput Human Behav. Pergamon; 2017;67: 84–94. doi: 10.1016/J.CHB.2016.10.014

[24] Mangen A, Schilhab T. An embodied view of reading: Theoretical considerations, empirical findings, and educational implications In: Matre s., Skaftun A, editors. Skriv! Les! Trondheim: Akademika forlag; 2012.

[25] Mizrachi D. Undergraduates’ Academic Reading Format Preferences and Behaviors. J Acad Librariansh. JAI; 2015;41: 301–311. doi: 10.1016/J.ACALIB.2015.03.009

[26] Zipf GK. Human behavior and the principle of least effort. Cambridge, Mass.: Addison-Wesley; 1949.

[27] Johnston N, Salaz AM, Alsabbagh L. Print and digital reading preferences and behaviors of University students in Qatar. Commun Comput Inf Sci. 2016;676: 247–255. doi: 10.1007/978-3-319-52162-6_24

[28] Landøy A, Repanovici A, Gastinger A. The more they tried it the less they liked it: Norwegian and Romanian student’s response to electronic course material. Commun Comput Inf Sci. 2015;552: 455–463.

[29] Terra A. Students’ reading behavior: digital vs. print preferences in Portuguese context. Commun Comput Inf Sci. 2015;552: 436–445.

[30] Zabukovec V, Vilar P. Paper or electronic: Preferences of Slovenian students. Commun Comput Inf Sci. 2015;552: 427–435.

[31] Pešut D, Živković D. Students’ academic reading format preferences in Croatia. New Libr World. 2016;117.

[32] Kortelainen T. Reading format preferences of Finnish university students. Commun Comput Inf Sci. 2015;552: 446–454.

[33] Tomczak M, Tomczak E. The need to report effect size estimates revisited. An overview of some recommended measures of effect size. Trends Sport Sci. 2014;1: 19–25.

[34] Cohen J. A power primer. Psychol Bull. 1992;112: 155 19565683 10.1037//0033-2909.112.1.155

[35] Porter SR, Whitcomb ME. Non-Response in Student Surveys: The Role of Demographics, Engagement and Personality. Res High Educ. 2005;46 doi: 10.1007/s11162-004-1597-2

[36] Harzing A-W, Brown M, Köster K, Zhao S. Response Style Differences in Cross-National Research. Manag Int Rev. SP Gabler Verlag; 2012;52: 341–363. doi: 10.1007/s11575-011-0111-2

[37] Si SX, Cullen JB. Response Categories and Potential Cultural Bias: Effects of an explicit middle point in cross-cultural surveys. Int J Organ Anal. MCB UP Ltd; 1998;6: 218–230. doi: 10.1108/eb028885

[38] Druckerman P. Books are Thriving in France. New York Times 10 7 2014.

[39] Anderson N. France attempts to impose e-book prices on Apple, others. Ars Technica 25 5 2011.

[40] Al Rawabdeh W, Zeglat D, Alzawahreh A. The Importance of Trust and Security Issues in E—Commerce Adoption in the Arab World. Eur J Econ. 2012;

